# Supplementary material for: Perceived exertion can be lower when exercising in field versus indoors
Source: PLoS One. 2024 May 29;19(5):e0300776. doi: 10.1371/journal.pone.0300776 (PMC11135770; doi:10.1371/journal.pone.0300776)
Supplement: S1 Appendix — (PDF) [file pone.0300776.s001.pdf]

S1 Appendix. Environmental description of the study by Ceci & Hassmén 1991.

Detailed descriptions of the used laboratory and outdoor field environments are necessary to comprehend how perceived exertion during physical exercise may be influenced by the external environment.

The study carried out by Ceci and Hassmén (1991) is noteworthy and well-controlled. However, a more thorough explanation of the laboratory and field environments involved could improve understanding of the surrounding conditions.

Through direct conversation in 2023 with Drs Ruggero Ceci and Peter Hassmén, independently of each other, Dr Peter Schantz established this description of the environmental settings of the study.

They both stated that the indoor running took place on a treadmill situated in a basement laboratory, where the runners faced a wall with no windows. Throughout the test, the only other person present in the room was the individual overseeing the test, aside from the runner.

The outdoor running occurred during the day on a trail in a natural setting beside a lake, featuring bare and smooth terrain, and in both spring and late autumn, regardless of leaf cover. Although single individuals may have passed the trail while running, they did not interrupt the activity.

The run began at the Academic Rowing Club and turned around at the Brunnsviken Canoe Club, located by Lake Brunnsviken in Stockholm, Sweden (Figure 1). Participants ran 250 meters in one direction before turning back towards the starting point. The running distance varied for different levels of rated perceived exertion (RPE), as detailed in the Methods section of Ceci and Hassmén (1991).

Figure 1 shows the laboratory location for treadmill running and the trail for outdoor running, while Figures 2-8 show ground based images of the trail utilized for outdoor runs.

Reference

Ceci, R. & Hassmén, P. 1991. Self-monitored exercise at three different RPE intensities in treadmill vs field running. Med Sci Sports Exerc 23(6):732-8.

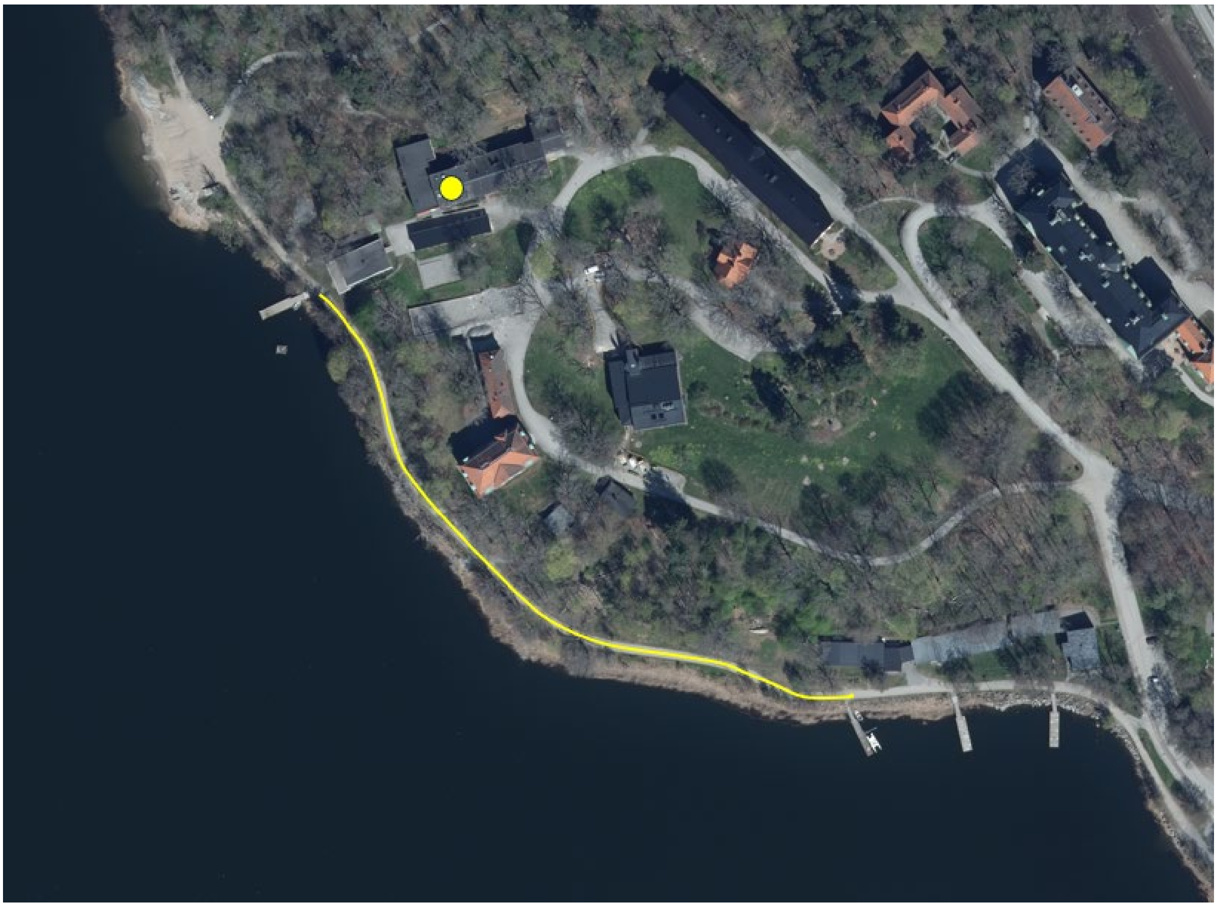

Figure 1. An aerial photo capturing Frescati Hage adjacent to Lake Brunnsviken in Stockholm, Sweden. A yellow dot marks the location of the RPE Research Unit, led by Professor Gunnar Borg, situated within the Department of Psychology at Stockholm University. This spot served as the venue for the laboratory and indoor running. The yellow line traces the trail along Lake Brunnsviken, utilized for field running. The setting has been conserved since 1986-87, when the data for the study was collected in comparison with the aerial photo taken in 2023. Photo: Copyright is granted from The Land Survey/Lantmäteriverket, ©Lantmäteriet, Gävle, Sweden, 2023.

The run started at the northern part of the trail where the Academic Rowing Club is located. Then the participants ran southeast along a curved trail, turned at the Brunnsviken Canoe Club, and ran back to the starting point at Academic Rowing Club, altogether 500 meters. The images below (Figure 2-8) illustrate the views that emerged when running along the trail from the canoe club to the rowing club. The photos in Figure 2-8 were all taken in 2009 and depict the same environmental conditions that existed when the study was undertaken in 1986-87.

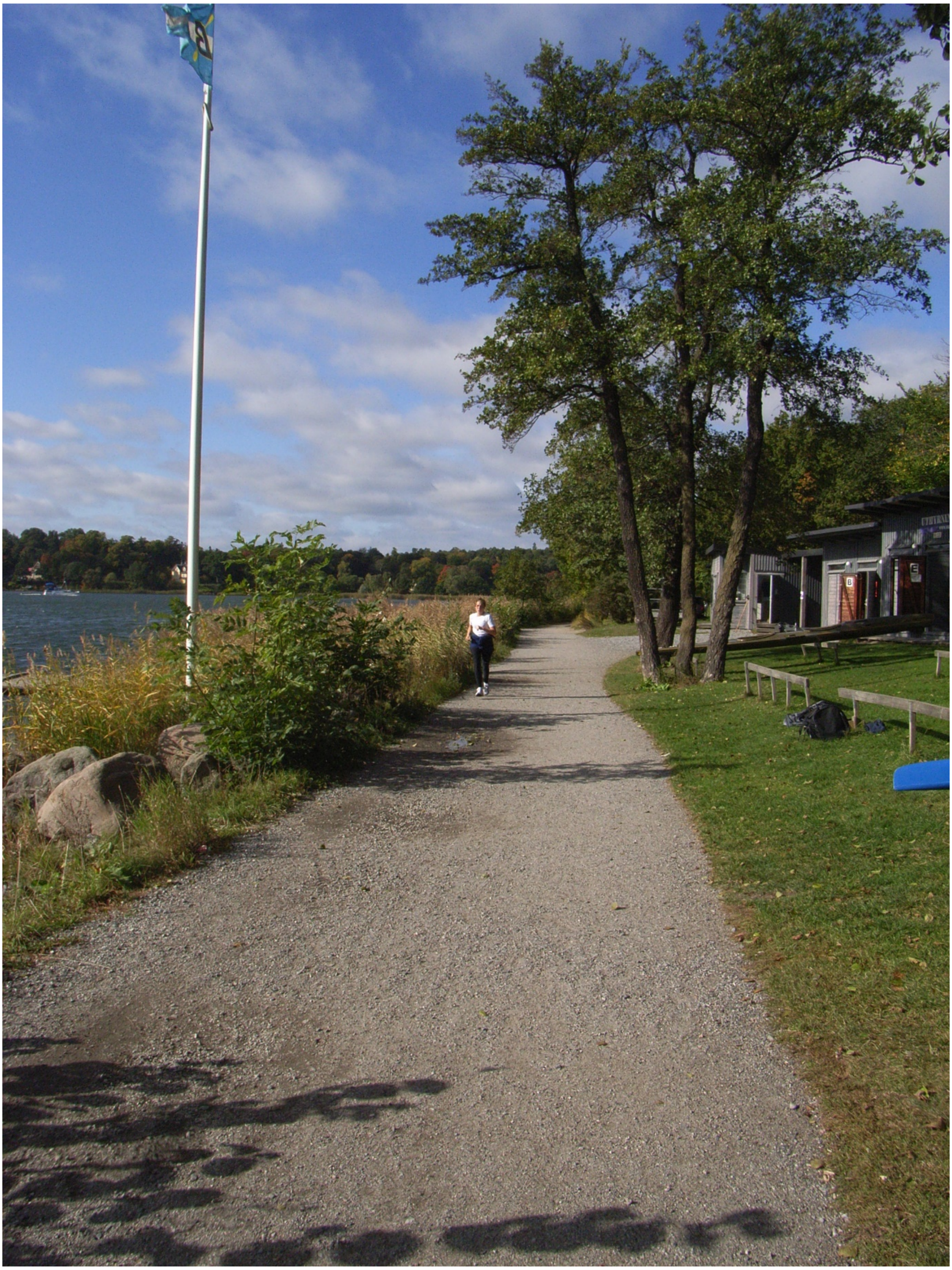

Figure 2. The turning point during the field running was located at the western end of the Brunnsviken Canoe Club to the right in the image. This point is located at the lake Brunnsviken, Stockholm, Sweden. Photo: Peter Schantz.

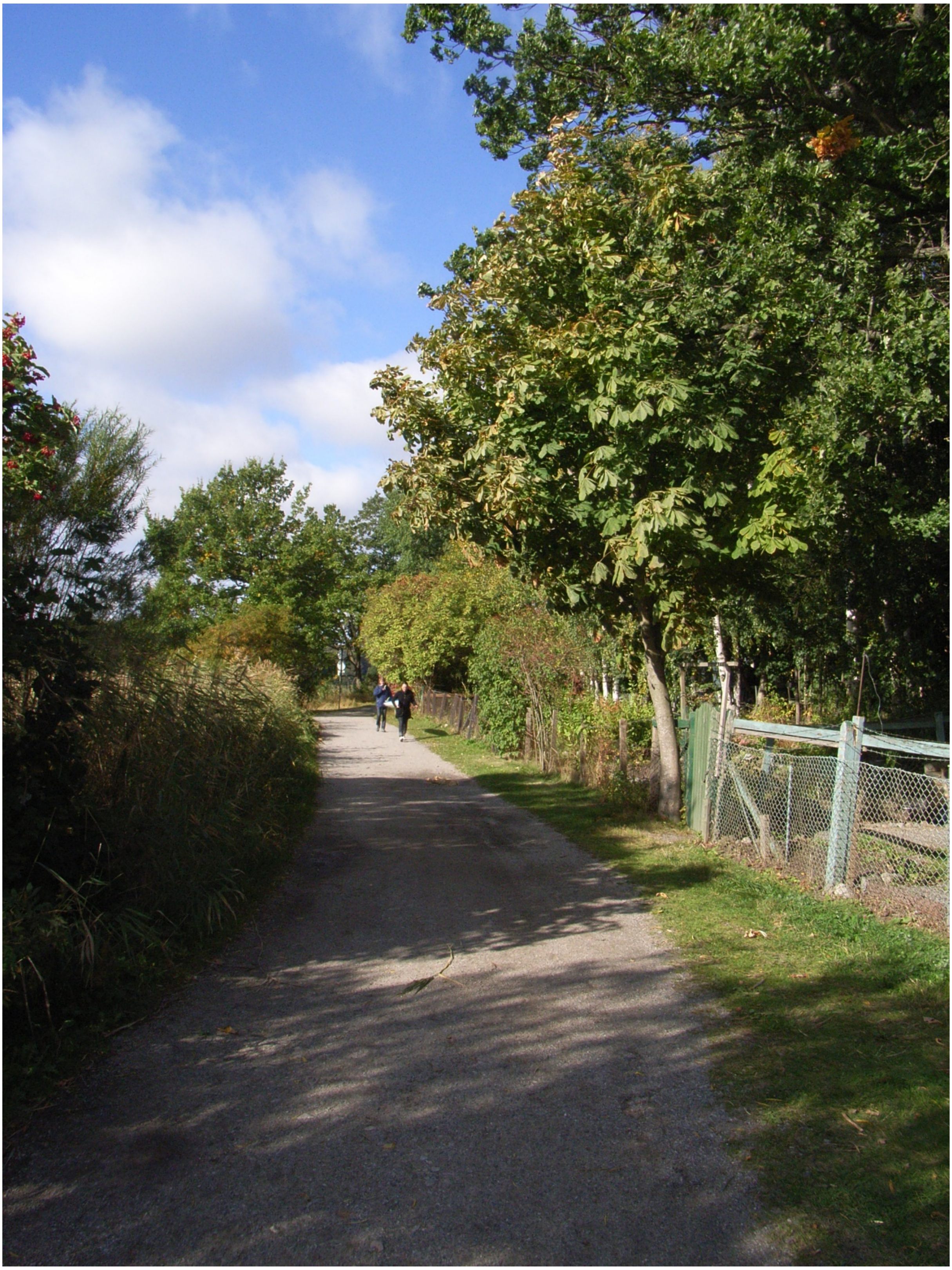

Figure 3. A consecutive part of the used running trail by the lake Brunnsviken, Stockholm, Sweden. Photo: Peter Schantz.

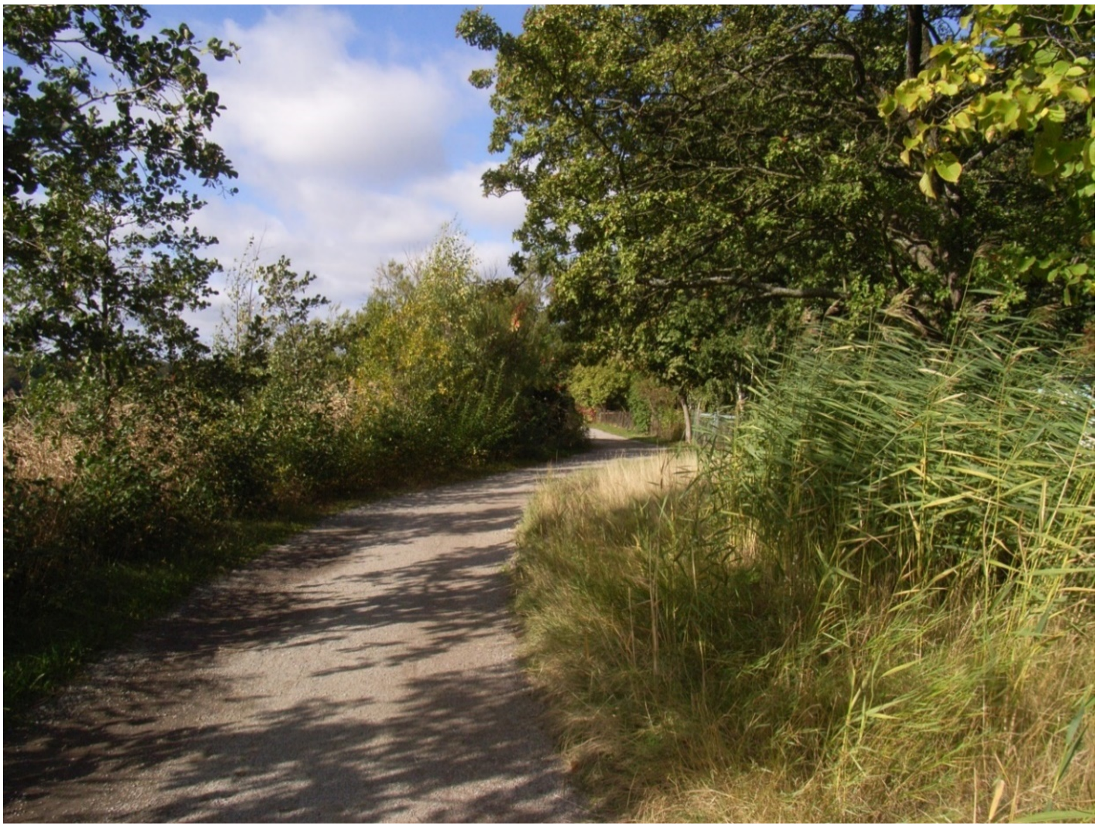

Figure 4. A consecutive part of used the running trail by lake Brunnsviken, Stockholm, Sweden. Photo: Peter Schantz.

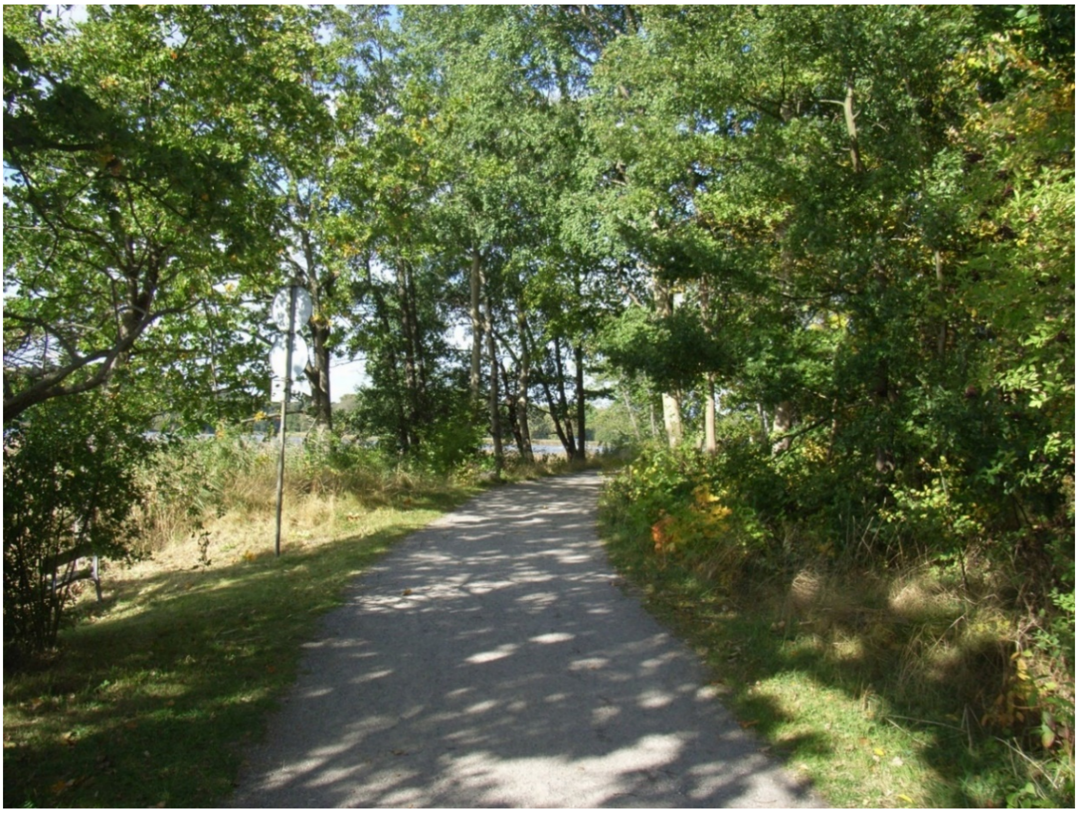

Figure 5. A consecutive part of the used running trail by lake Brunnsviken, Stockholm, Sweden. Photo: Peter Schantz.

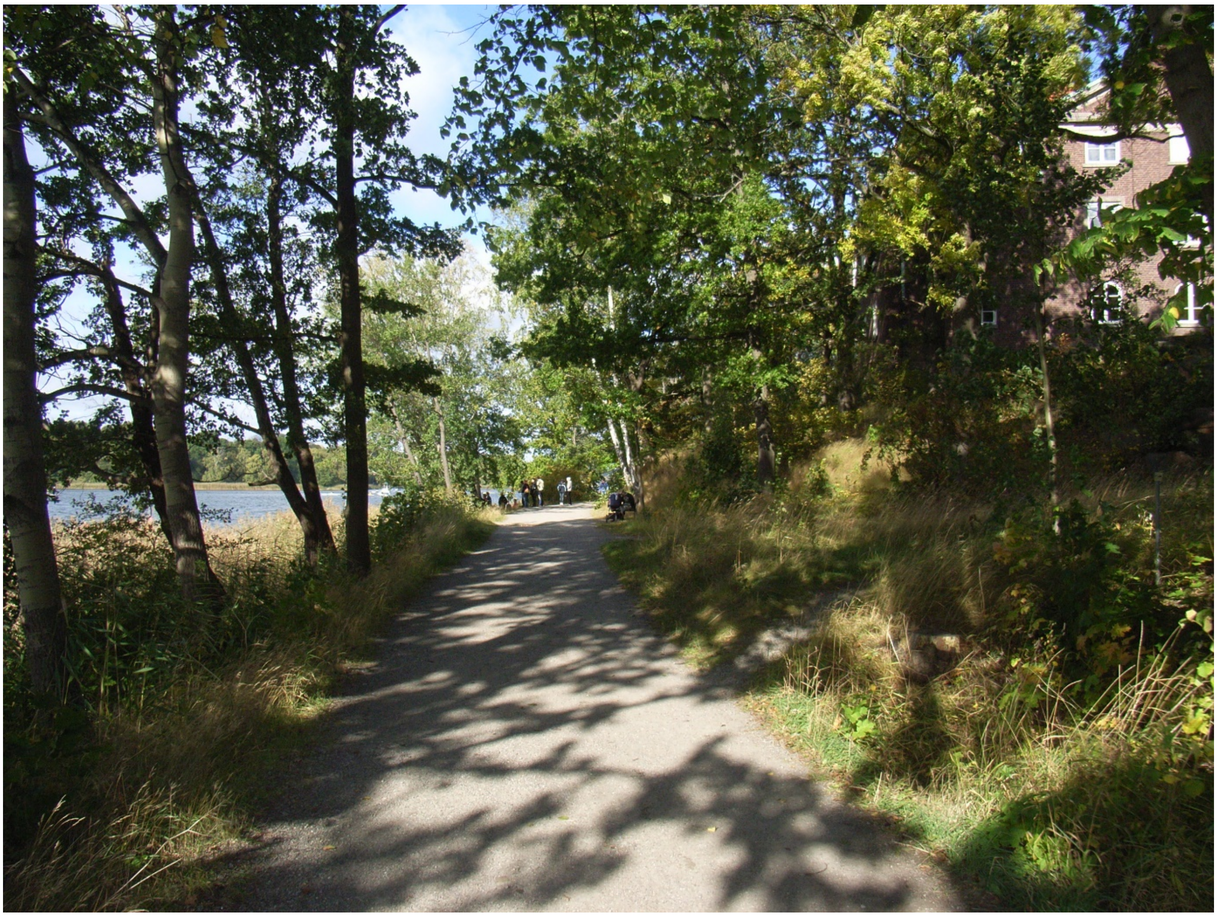

Figure 6. A consecutive part of the used running trail by lake Brunnsviken, Stockholm, Sweden. Photo: Peter Schantz.

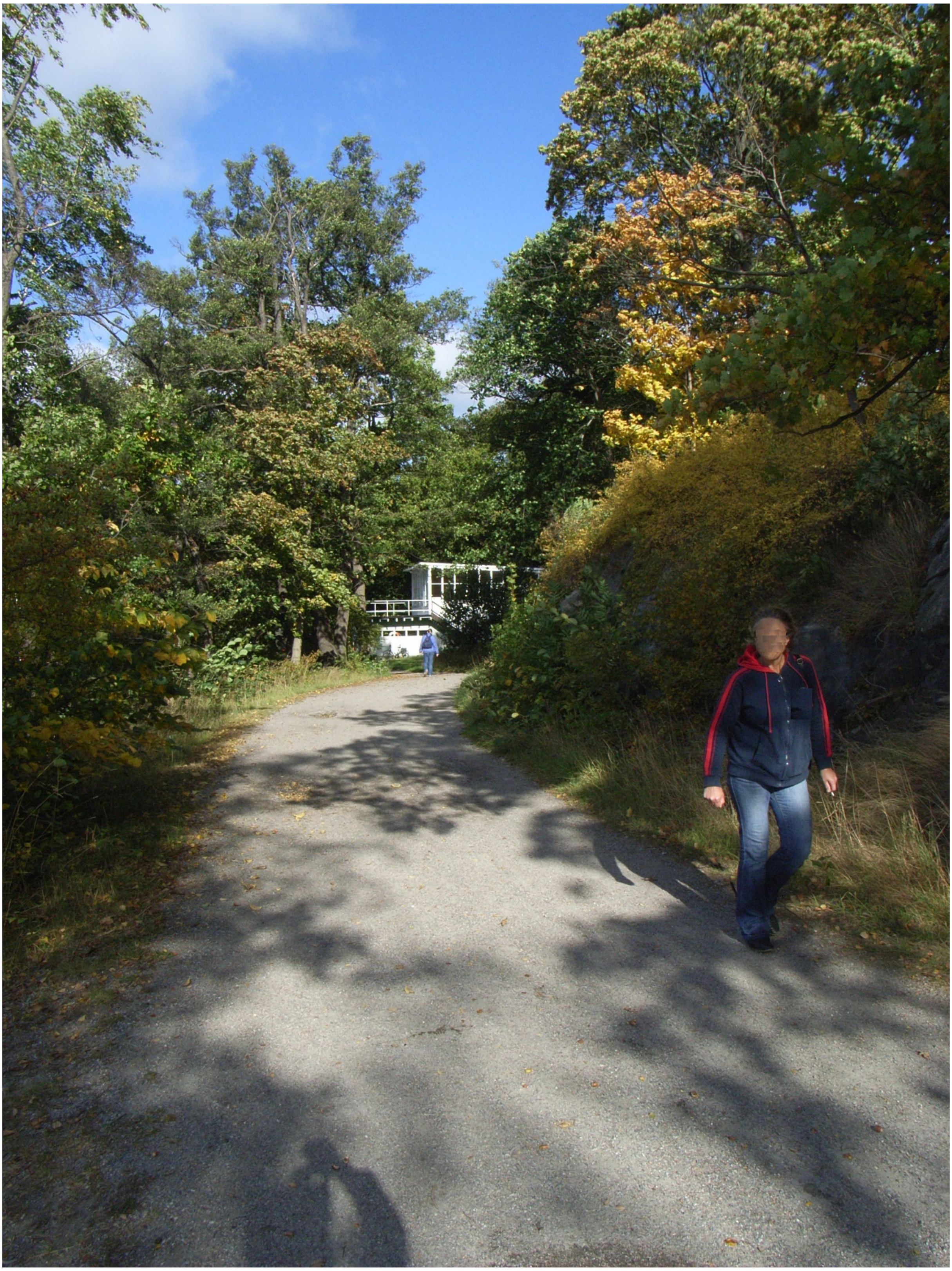

Figure 7. The view from the running trail, south of the Academic Rowing Club, by lake Brunnsviken in Stockholm, Sweden. Photo: Peter Schantz.

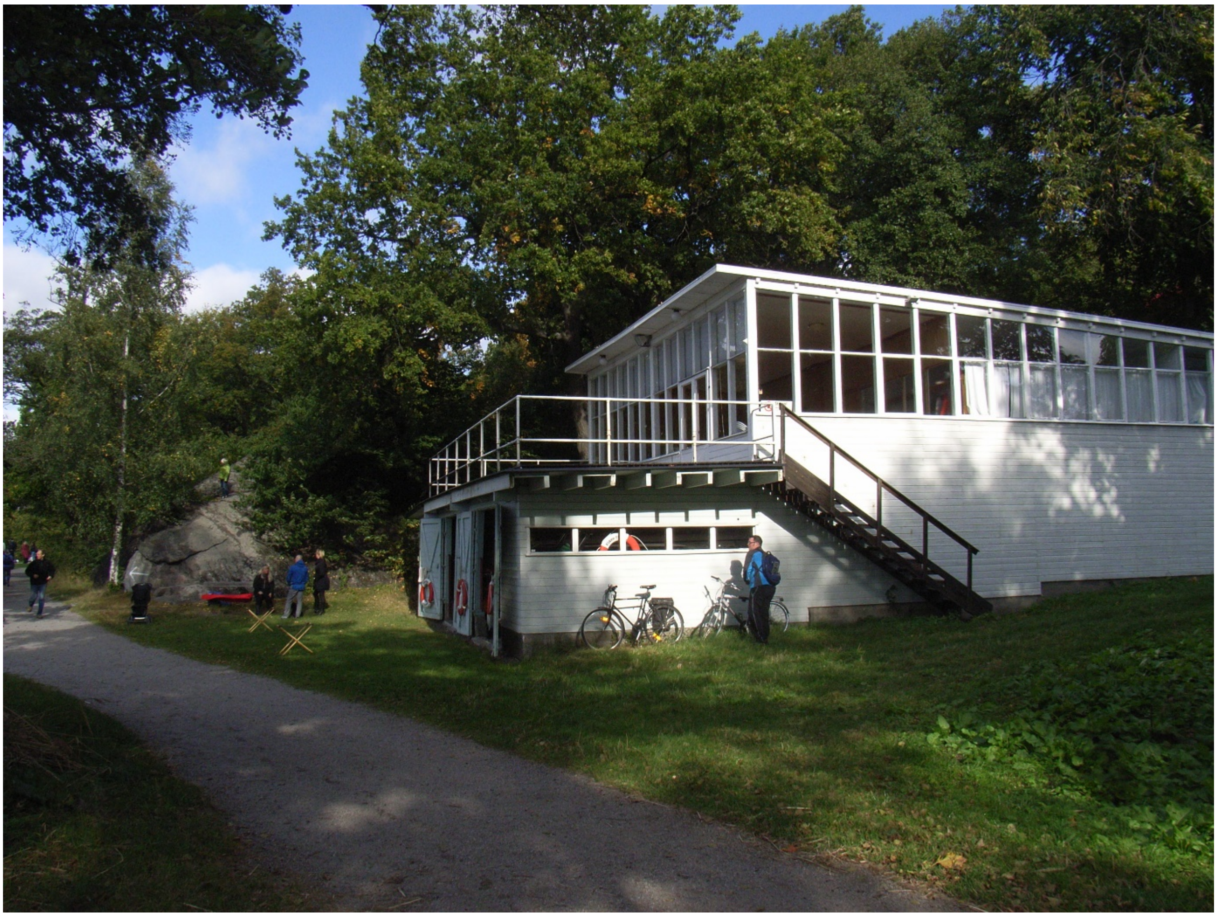

Figure 8. The starting point for the field running at the Academic Rowing Club by lake Brunnsviken in Stockholm, Sweden. Photo: Peter Schantz.
