# Supplementary material for: Perceived exertion can be lower when exercising in field versus indoors
Source: PLoS One. 2024 May 29;19(5):e0300776. doi: 10.1371/journal.pone.0300776 (PMC11135770; doi:10.1371/journal.pone.0300776)
Supplement: S2 Appendix — (PDF) [file pone.0300776.s002.pdf]

S2 Appendix. Environmental description of the study by Mieras et al. 2014.

To understand if the external environment may influence the rated perceived exertion during physical activity, the descriptions of the laboratory and the external environments are important.

The study by Mieras et al. (2014) is well-developed in its strategy and important in that respect. The aim was to compare self-paced physical exercise outdoors *vs* indoors from several perspectives. The outdoor setting for cycling was the Keystone Trail in Omaha, Nebraska.

The descriptive matters of the settings along the Keystone Trail have been furthered by Dr Peter Schantz in a dialogue with the corresponding author, Dr. Dustin Slivka, and the landscape architect Dennis Bryers, at the Park Planning Division, Omaha Parks, Recreation & Public Property Department, City of Omaha, in 2024.

First, when the participants cycled indoors, it was in a laboratory, and they viewed a wall without any window.

The Keystone Trail runs through the Omaha metropolitan area in Nebraska, USA. It follows a stream named Papillion Creek. It passes through residential areas, commercial/retail areas, and farmland.

20 km of this trail was used in both directions, creating altogether 40 km. The starting point was close to Western Avenue in the north, and the turning point was close to the West Papio intersection in the south (Figure 1-3).

This is a smooth paved trail, often about 6 ft wide. It is mostly rather straight, with a few turns to cross the creek over bridges. It is also mostly flat, with a few small underpasses that dip down and then up. The trail is framed in various ways (see below).

During the test for the study, there were other cyclists and pedestrians on the trail. The trail is, however, wide enough that a cyclist can easily pass without much wait. Therefore, cyclists were not impeded by either other cyclists or pedestrians and were able to hold their power steady.

In Figure 1-3 follows three aerial images showing the parts of the Keystone Trail used, from north to south. These images depict rather current conditions. The study using the Keystone Trail was undertaken in 2013, while the aerial photos in Figure 1-3 were taken in about 2023. However, according to landscape architect Dennis Bryers, at the City of Omaha, very few and minor changes have been undertaken along the trail between the time for the study and when the photos were taken.

Reference

Mieras ME, Heesch, MWS & Slivka, DR. 2014. Physiological and psychological responses to outdoor vs. laboratory cycling. J Strength Cond Res 28(8):2324-9.

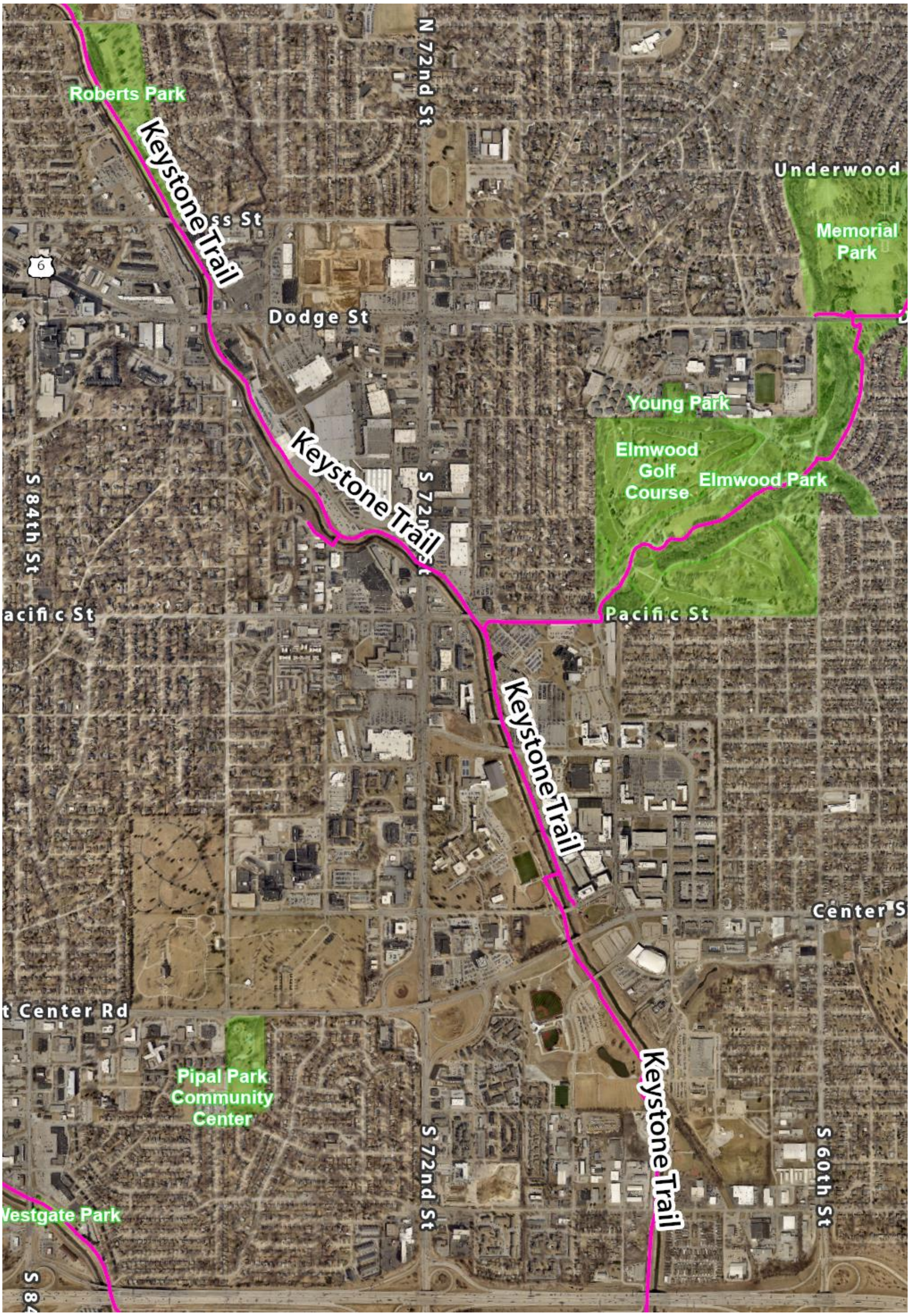

Figure 1. The Keystone Trail in Omaha, Nebraska, USA, follows Papillion Creek, and in this image, it runs diagonally. The starting point for the cycling was close to Western Avenue, northeast of the University of Nebraska Omaha. Photo: Open source via Douglas County GIS, Nebraska. For map questions, contact: gis@douglascounty-ne.gov

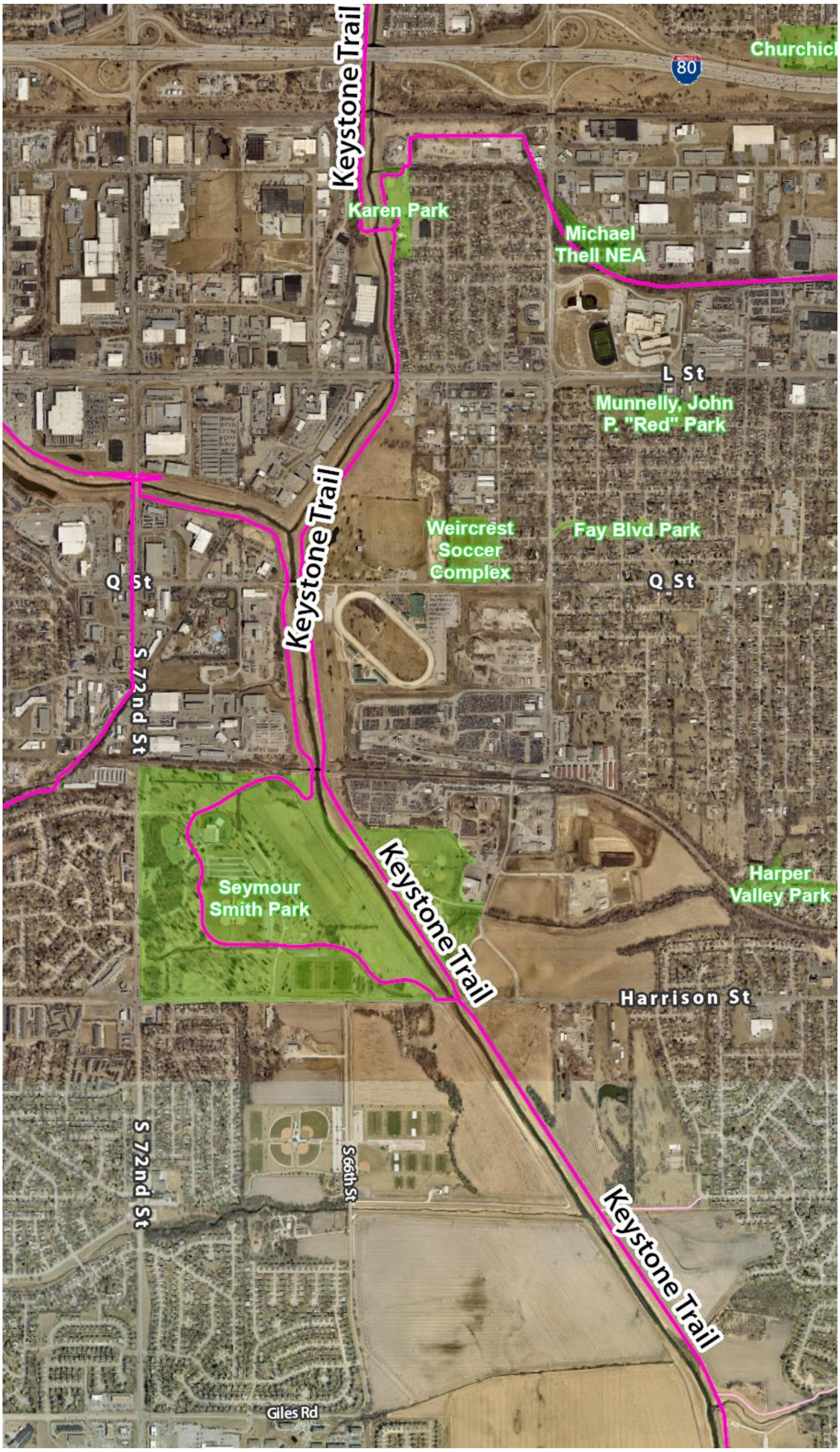

Figure 2. The Keystone Trail in Omaha, Nebraska, USA, follows Papillion Creek, and here we see a section in which it runs mainly in farmland. Photo: Open source via Douglas County GIS, Nebraska. For map questions, contact: gis@douglascounty-ne.gov

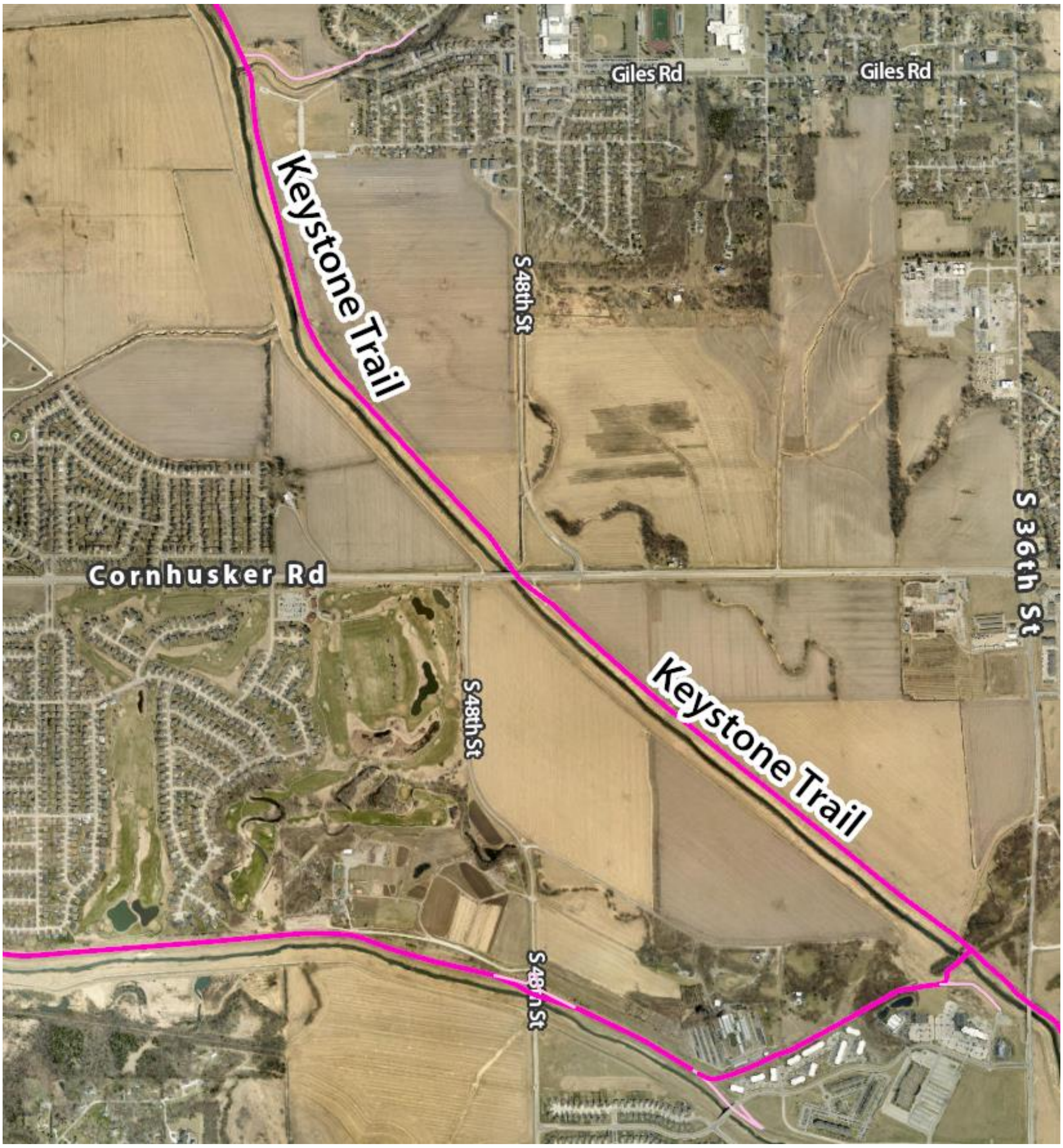

Figure 3. The Keystone Trail in Omaha, Nebraska, USA, follows Papillion Creek, and here we see a section in which it runs in mainly farmland. The turning point for the cycling was in the lower right corner where the West Papio intersection is located. Photo: Open source via Douglas County GIS, Nebraska. For map questions, contact: gis@douglascounty-ne.gov

Based on aerial photos from Google Earth in magnified versions, and close-up street views, it was noted that the cyclists passed through three rather distinctly different kinds of areas in terms of predominant land use: (i) residential areas, (ii) commercial/retail areas, and (iii) farmland.

The distance proportions for these types of settings were about 5% residential areas, 50% commercial/retail areas, and 45% farmland. Some parts of the commercial/retail areas did, however, have lines of trees between them and the Keystone Trail, and sometimes residential areas were located

on the opposite side of the creek. The farmland was mostly open but was sometimes framed with trees and/or bushes. Adjacent to the Papillion Creek were green slopes along the whole cycle ride. Thus, although the surroundings of the Keystone Trail differ a lot along the ride, the creek and its immediate green surroundings remained a stable feature.

To illustrate what these settings looked like, see figures 4-17 below. Finally, note that the cycling took place in a part of the year when the landscape was green, and with e.g., leaves still in the trees.

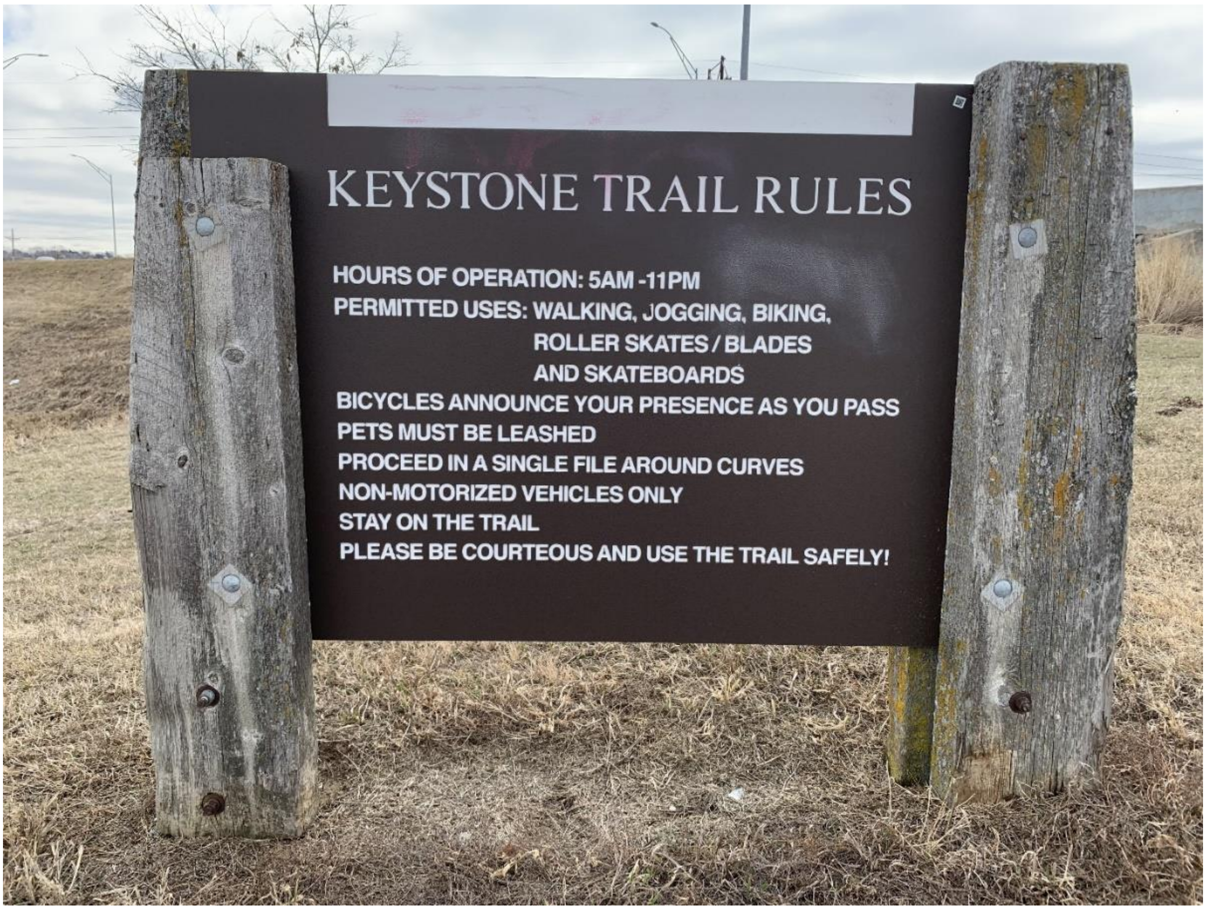

Figure 4. The rules for Keystone Trail in Omaha, Nebraska, USA. Photo: Dennis E. Bryers, FASLA, PLA

Representative examples of residential areas framing the Keystone Trail

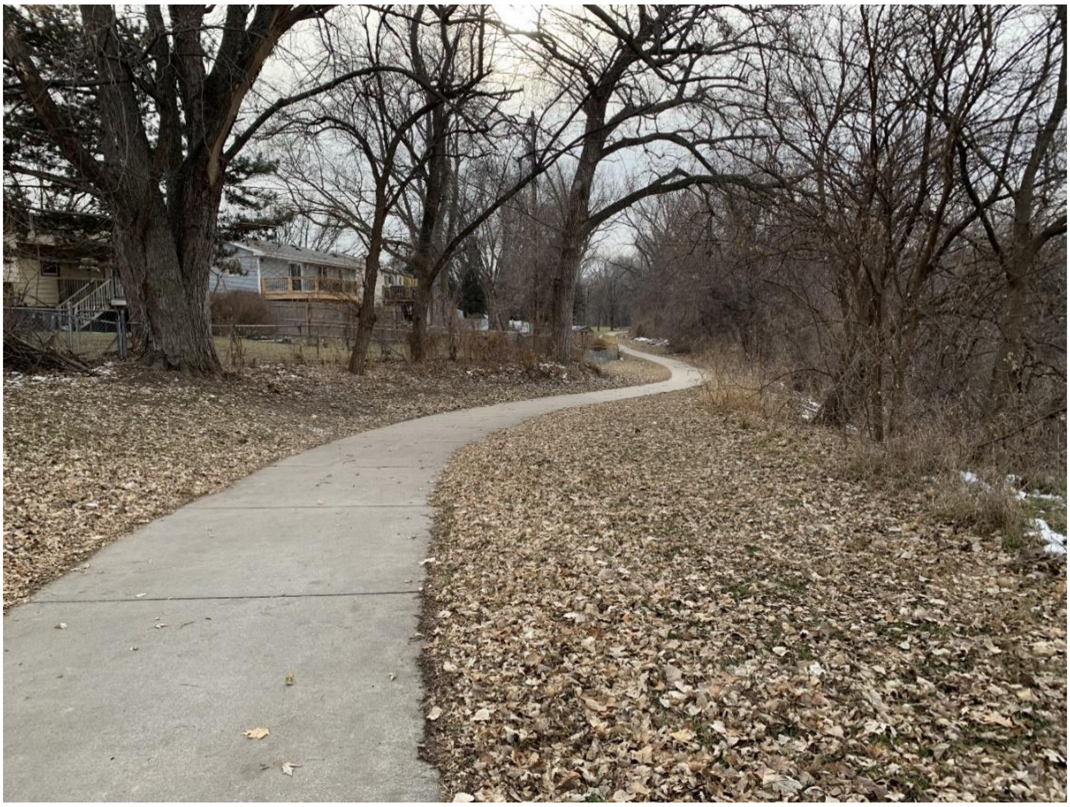

Figure 5. Example of residential setting along the Keystone Trail in Omaha, Nebraska, USA. Photo: Dennis E. Bryers, FASLA, PLA

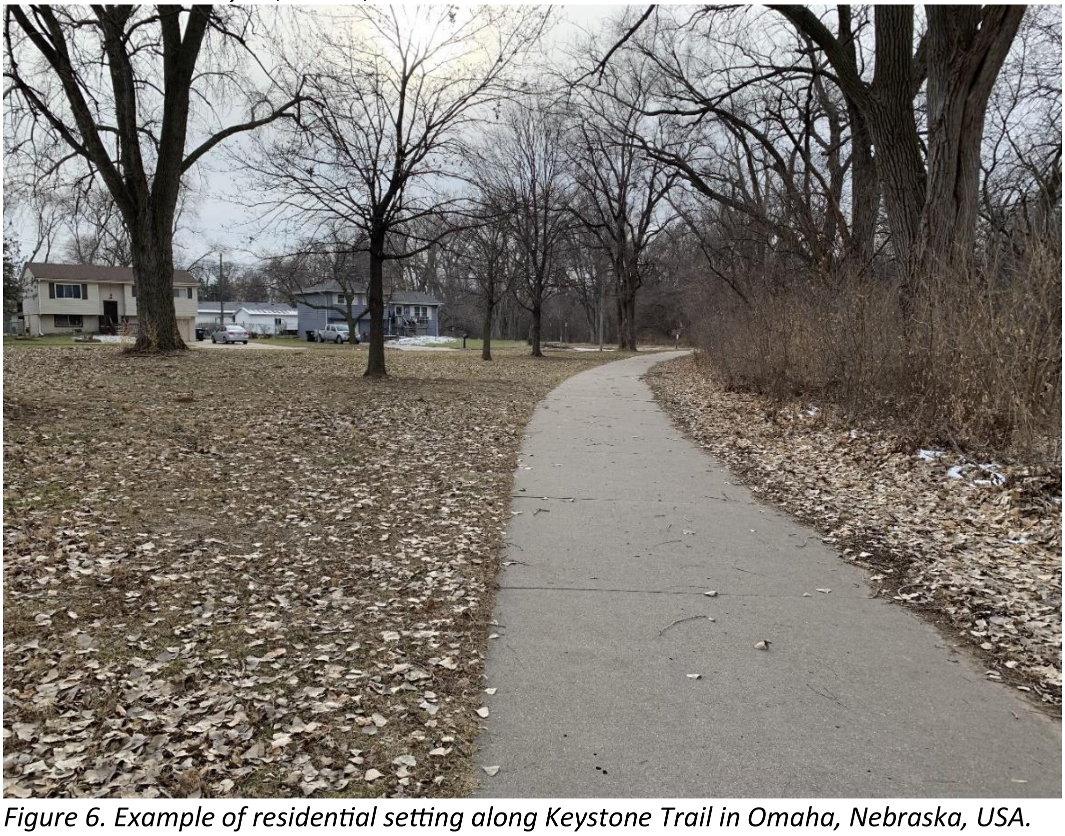

Figure 6. Example of residential setting along Keystone Trail in Omaha, Nebraska, USA. Photo: Dennis E. Bryers, FASLA, PLA

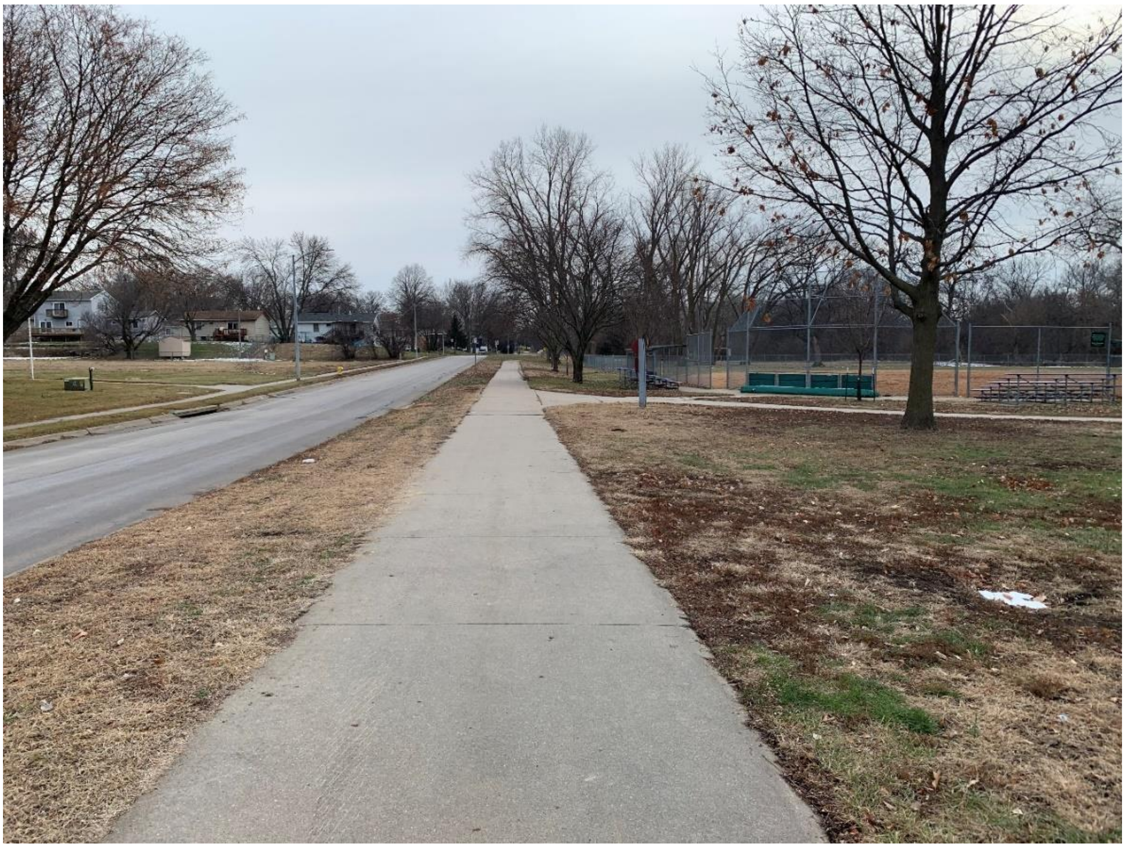

Figure 7. Example of residential setting along Keystone Trail in Omaha, Nebraska, USA. Photo: Dennis E. Bryers, FASLA, PLA

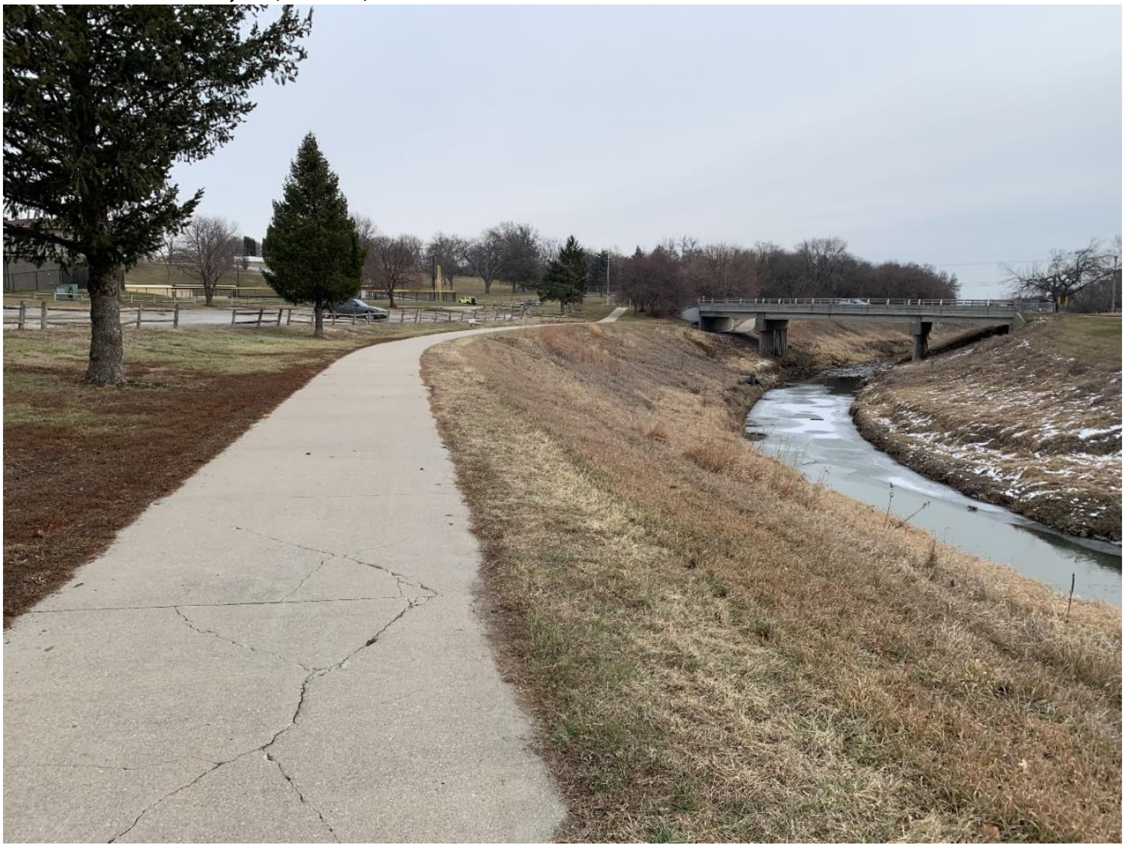

Figure 8. Example of residential setting along Keystone Trail in Omaha, Nebraska, USA. Photo: Dennis E. Bryers, FASLA, PLA

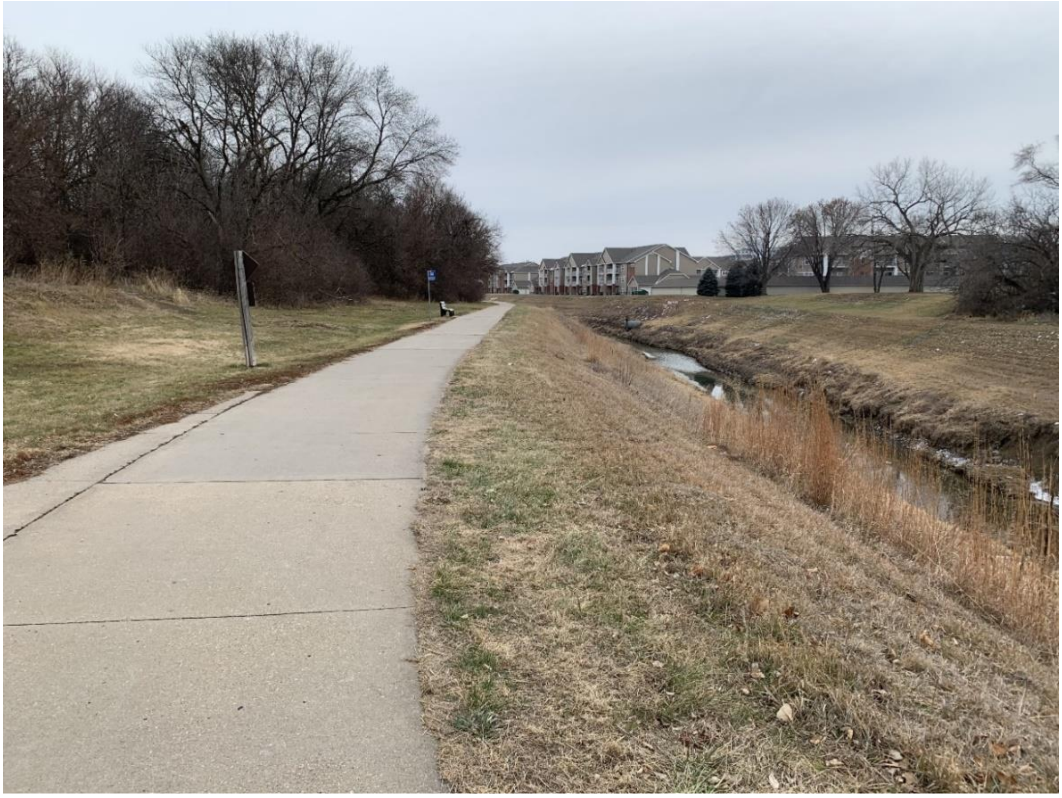

Figure 9. Example of residential setting along Keystone Trail in Omaha, Nebraska, USA. Photo: Dennis E. Bryers, FASLA, PLA

Representative examples of commercial/retail areas framing the Keystone Trail

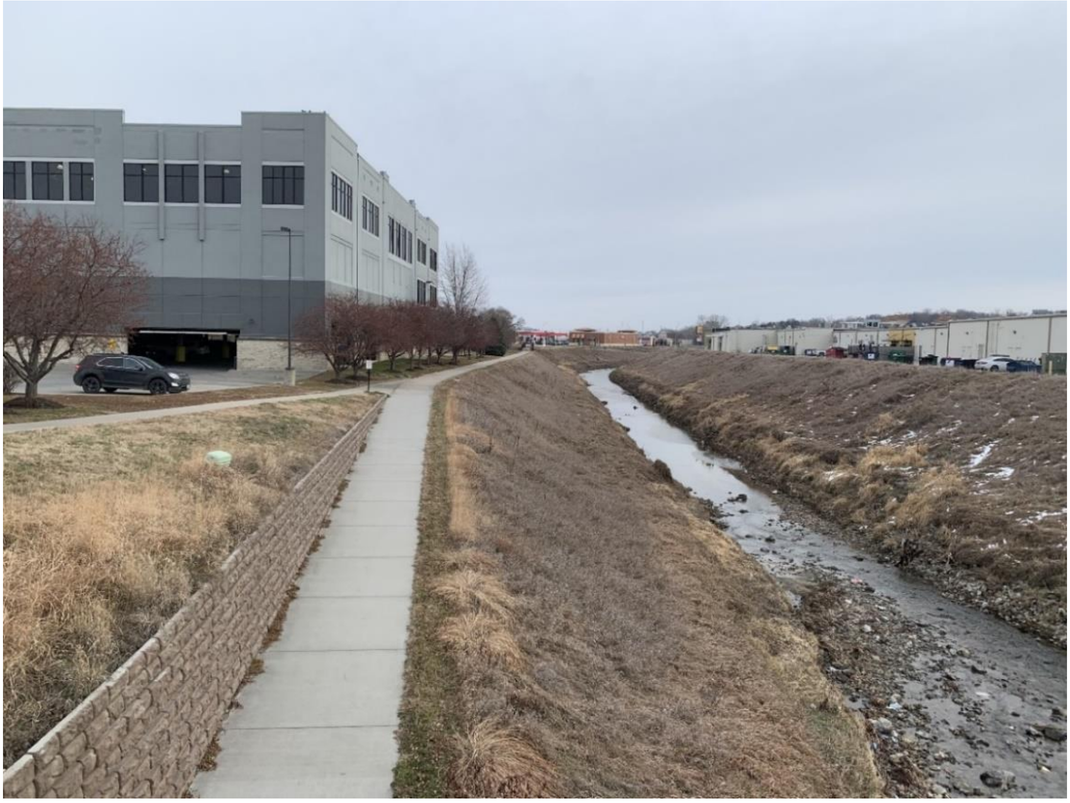

Figure 10. Example of commercial or retail setting along Keystone trail in Omaha,

Nebraska, USA. Photo: Dennis E. Bryers, FASLA, PLA

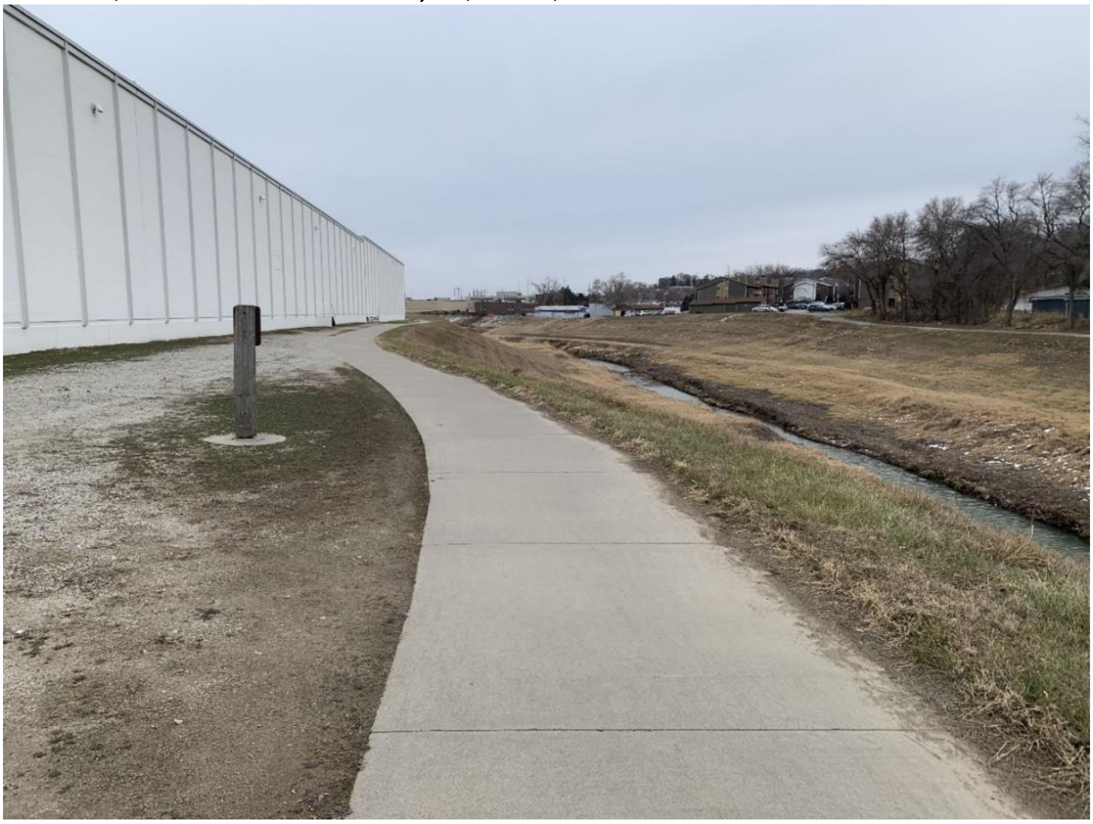

Figure 11. Example of commercial or retail setting along Keystone Trail in Omaha, Nebraska, USA. Note, however, that on the other side of the creek, the framing of it is rather green. Photo: Dennis E. Bryers, FASLA, PLA

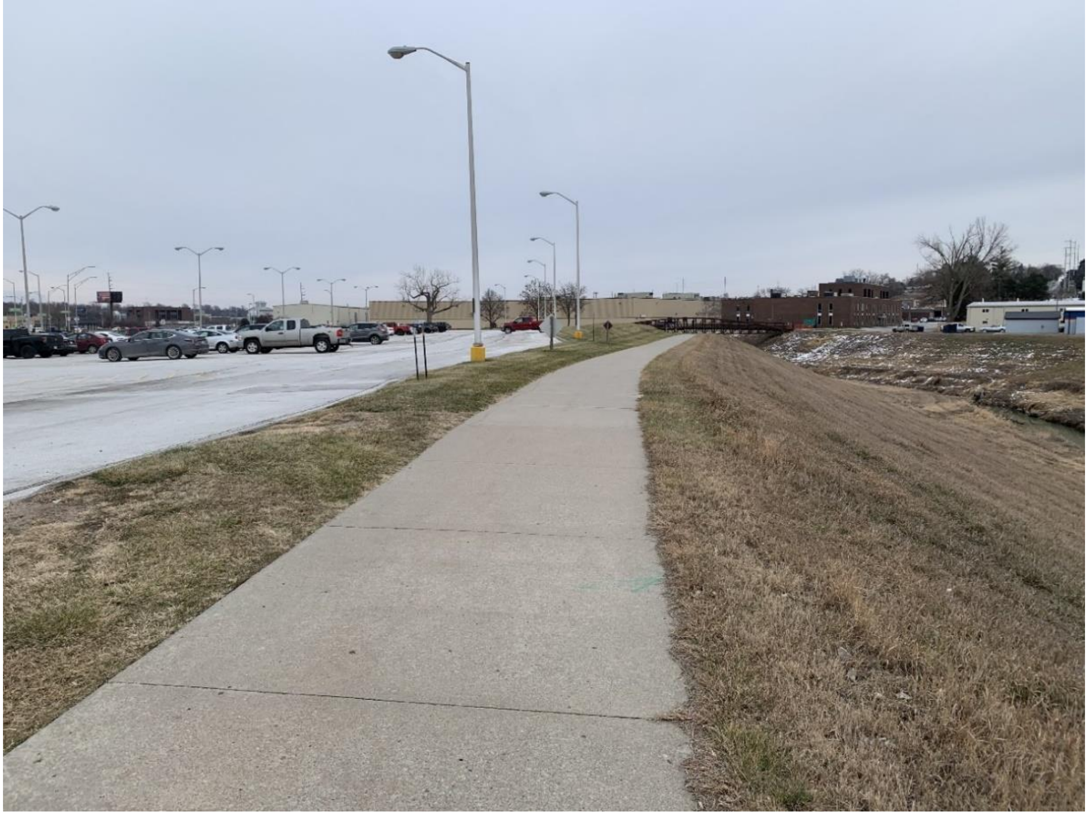

Figure 12. Example of commercial or retail setting with a parking lot along Keystone Trail in Omaha, Nebraska, USA. Photo: Dennis E. Bryers, FASLA, PLA

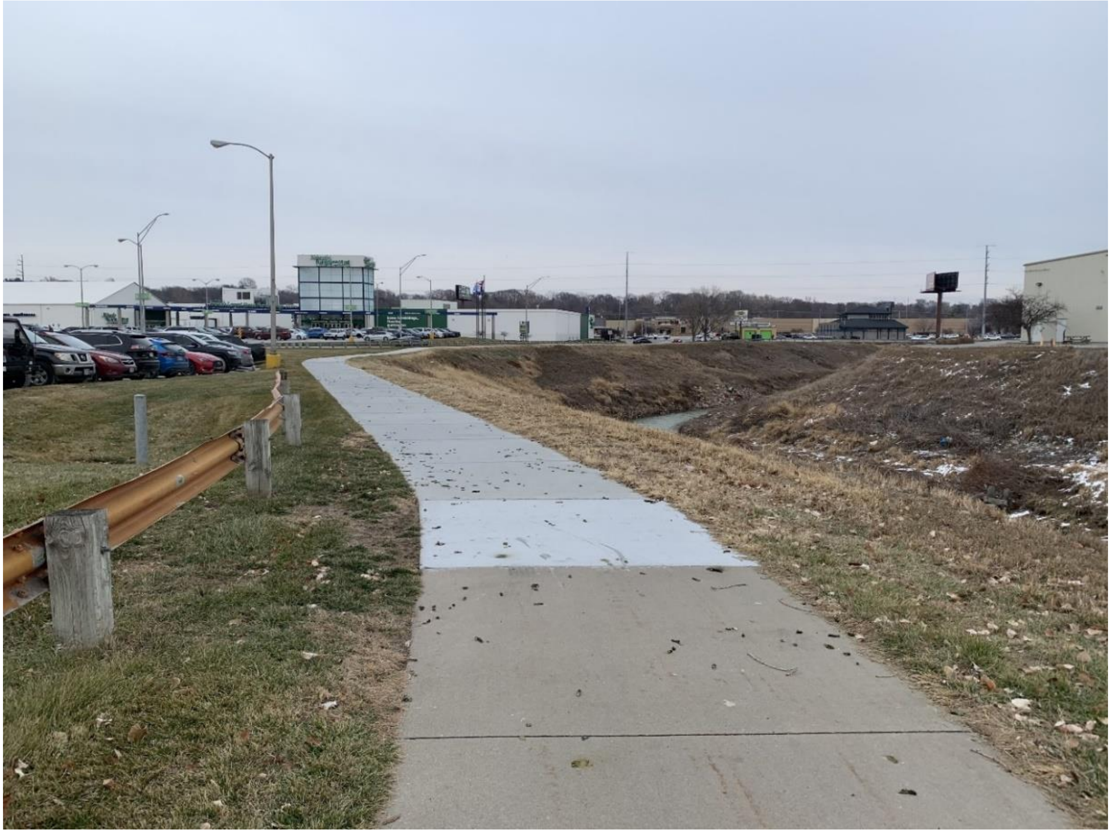

Figure 13. Example of commercial or retail setting with a parking lot along Keystone Trail in Omaha, Nebraska, USA. Photo: Dennis E. Bryers, FASLA, PLA

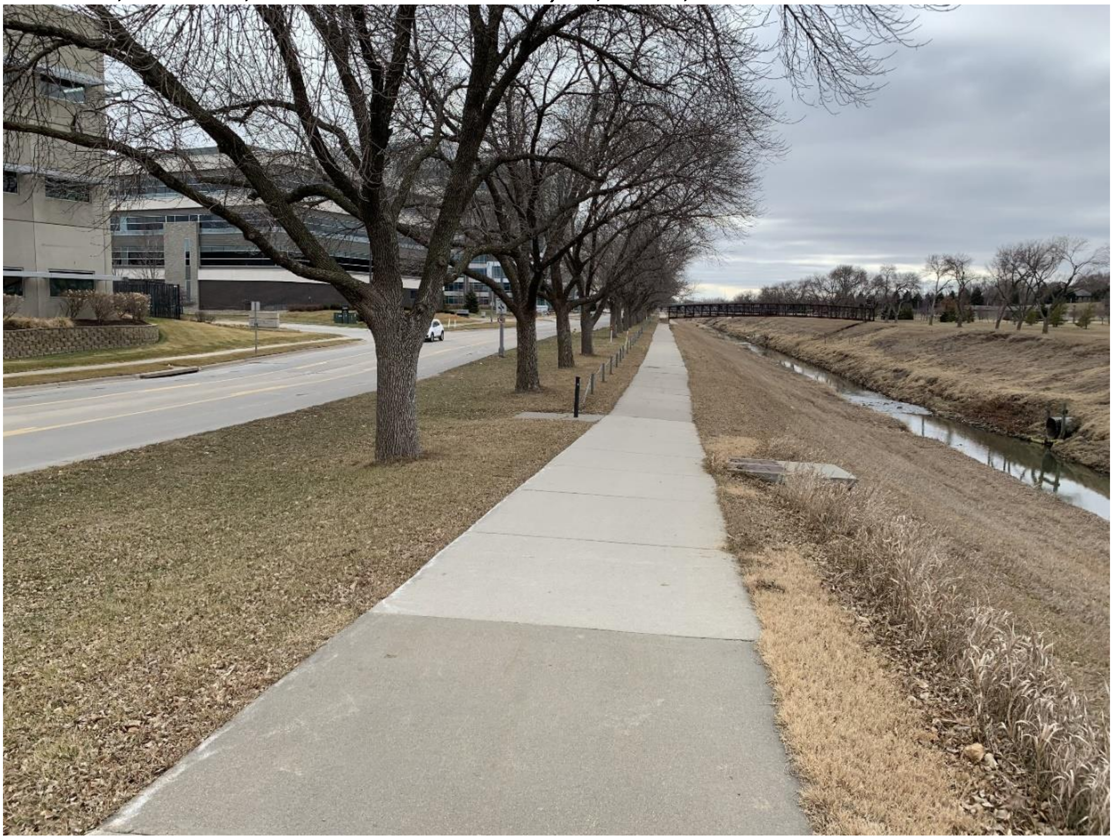

Figure 14. Example of an office setting along Keystone Trail in Omaha, Nebraska, USA. Photo: Dennis E. Bryers, FASLA, PLA

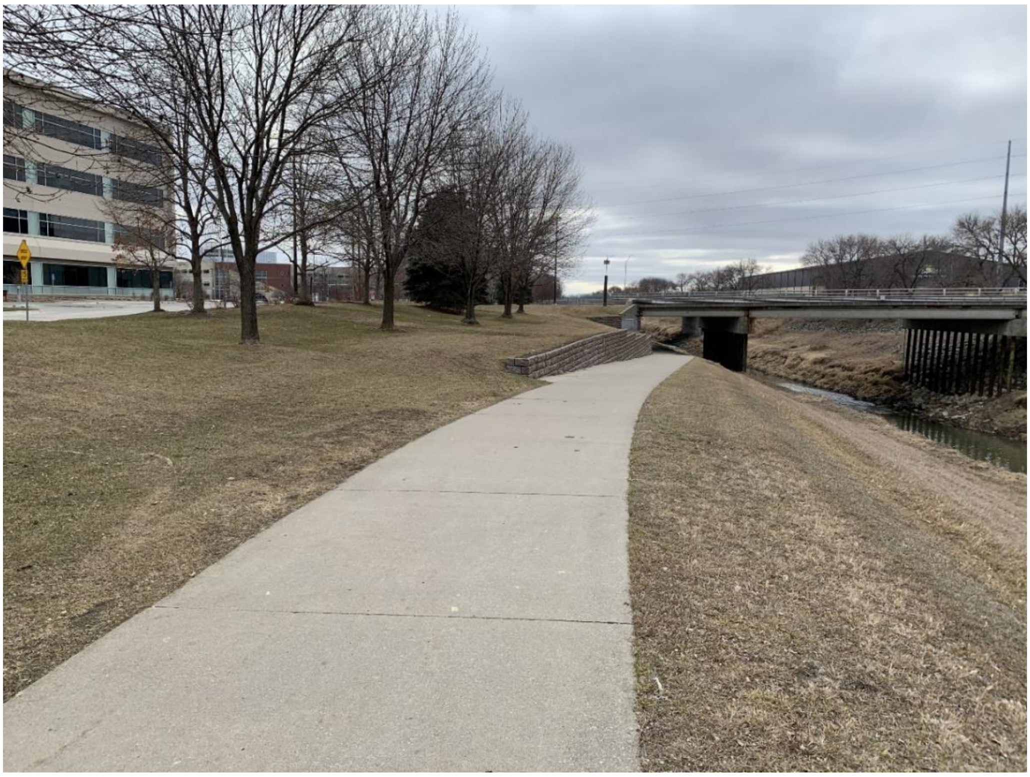

Figure 15. Example of an office setting along Keystone Trail in Omaha, Nebraska, USA. The image also shows the uncoupling between the trail and the infrastructure for motorized traffic. Photo: Dennis E. Bryers, FASLA, PLA

Representative examples of farmland areas framing the Keystone Trail

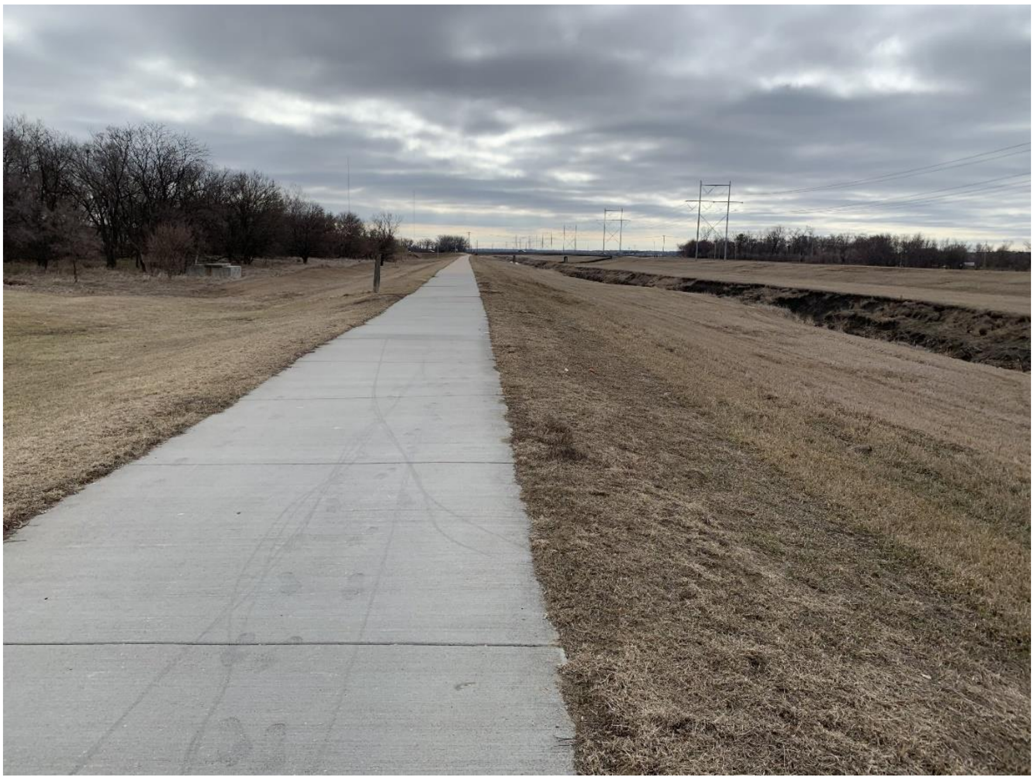

Figure 16. Example of a rural setting along Keystone Trail in Omaha, Nebraska, USA. Photo: Dennis E. Bryers, FASLA, PLA

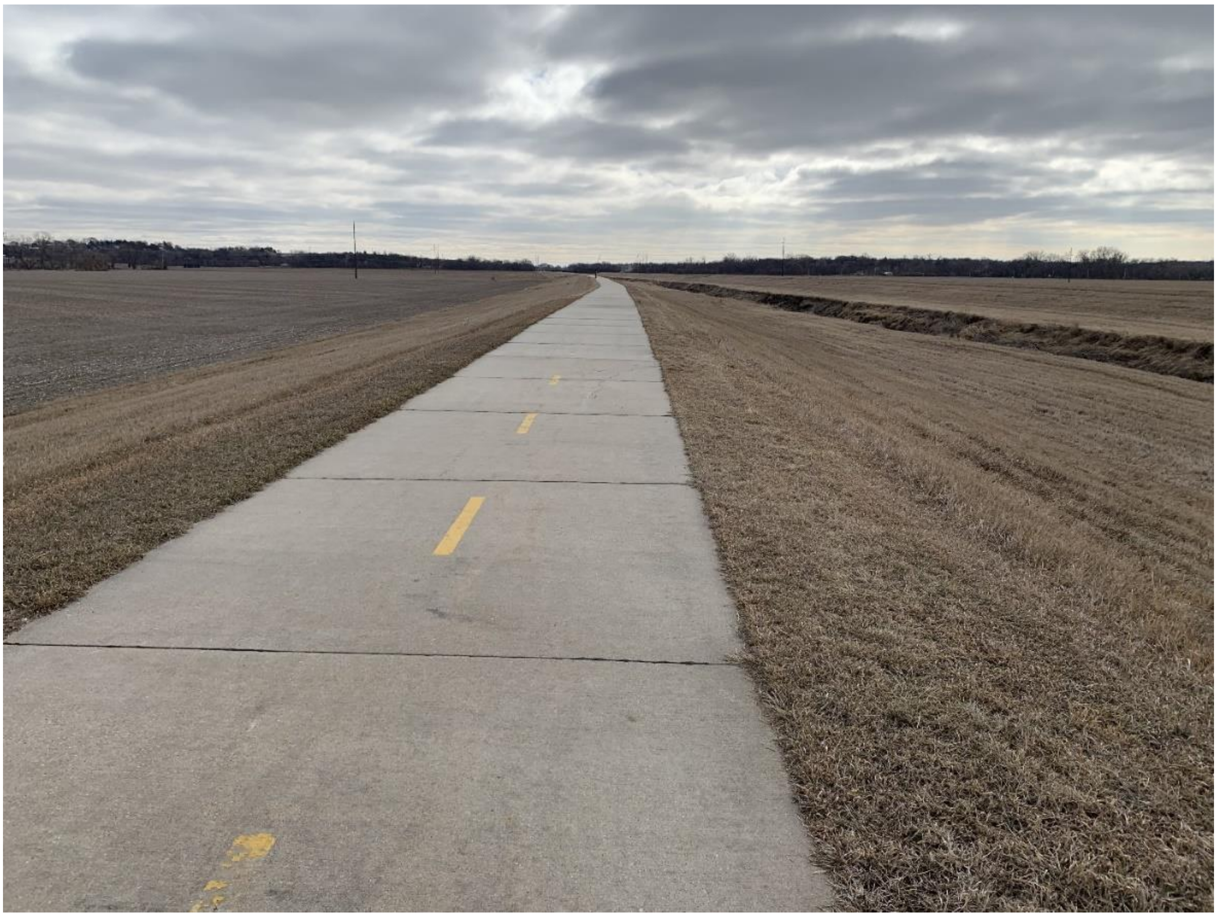

Figure 17. Example of a rural setting along Keystone Trail in Omaha, Nebraska, USA.
Photo: Dennis E. Bryers, FASLA, PLA
