# Supplementary material for: Perceived exertion can be lower when exercising in field versus indoors
Source: PLoS One. 2024 May 29;19(5):e0300776. doi: 10.1371/journal.pone.0300776 (PMC11135770; doi:10.1371/journal.pone.0300776)
Supplement: S3 Appendix — (PDF) [file pone.0300776.s003.pdf]

S3 Appendix. Environmental description of the present study by Olsson et al. 2024.

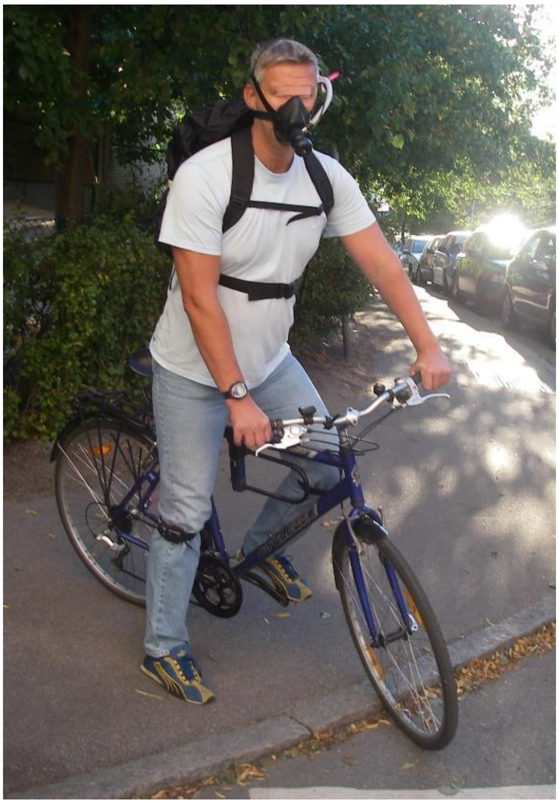

In the laboratory and during the cycle commuting, metabolic measurement systems were used. Both systems used a mask as illustrated in Figure 1, whereas in the field setting the measurement system was mobile and carried on the back by the cyclists.

Figure 1. A participant with a mobile metabolic measurement system, and ready to start his commuter cycling trip. Photo: Peter Schantz.

All participants had drawn their cycling routes on a map (see example in Figure 2). In this way, as well as through the test leaders' visits to the points of origin and destination for each cycling trip, and through driving a car between these places, the characteristics of the settings cycled in were noted. Furthermore, it was noted where the trips took place in relation to the distinction between the inner urban and rural-suburban parts of the metropolitan area of Greater Stockholm (cf. Figure 3). Based on that knowledge, it is clear that every cyclist experienced a very stimulus-rich environment during the commute.

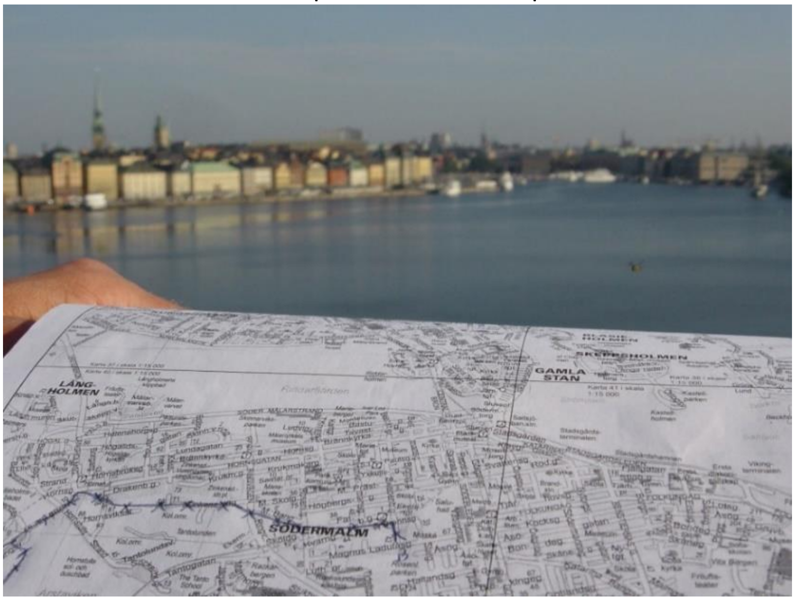

Figure 2. Every participant drew their cycling commuting route on a map such as the one illustrated here. The image is from the center of the inner urban part of Stockholm, with the Old Town to the left. In this specific map, however, the route marked was taken by a commuting pedestrian. Photo: Peter Schantz.

A map showing the overall study area in Stockholm County, Sweden, with the exception being the Norrtälje Municipality (Figure 3). All the cycling trips studied took place in the suburban and/or inner urban parts of the study area.

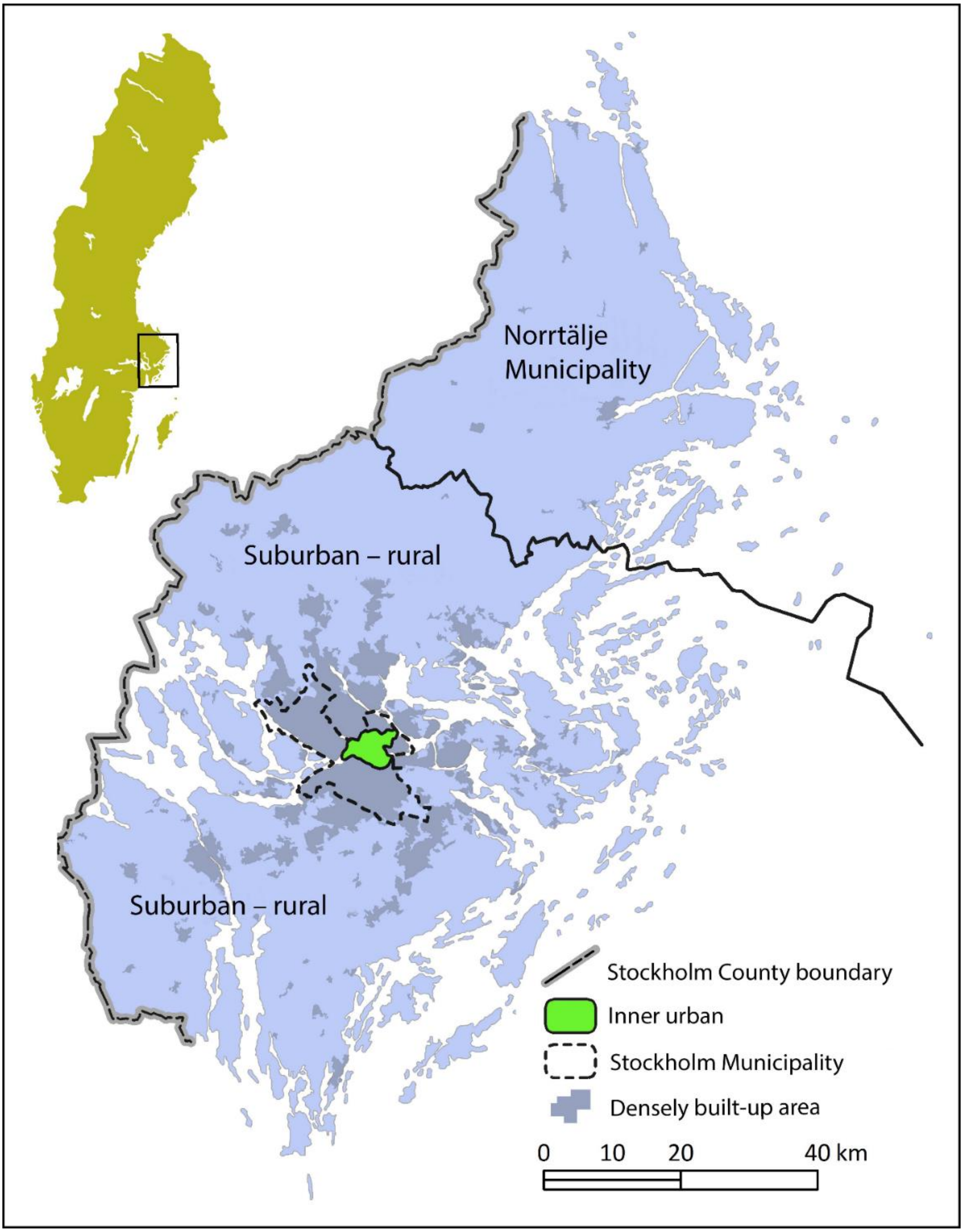

Figure 3. The study area was the County of Stockholm, Sweden, except for the Norrtälje municipality. Given the very different environmental characters of the inner urban parts of the Municipality of Stockholm, and the suburban and rural parts of the remainder of the county, a distinction was made between them when the cyclists rated their route environmental characteristics (see next page). Map: Magnus Strömgren.

Descriptions of the external environment are important to understand how the environment can influence the perceived exertion during physical activity. Given that in this study, 20 different cycling routes were taken in different parts of the study area, this is a difficult task.

In the main text for the study, we describe the average ratings for the trips in terms of the cyclist's appraisals of whether their cycling routes are overall hindering – stimulating for the cycling, as well as unsafe – safe for reasons of traffic. The variability in the route environments concerning both single and combinations of environmental variables as well as in the urban and suburban settings has been described in more detail by Wahlgren and Schantz (2011). In it, the study area is also described in more detail in other ways.

Here we will indicate the characteristics of the study area by showing an inner urban setting with its typically about 5-6 stories high buildings in blocks and grid-like road systems (Figure 4). To further visualize the character of the study area, two aerial photographs are included, see Figures 5 and 6.

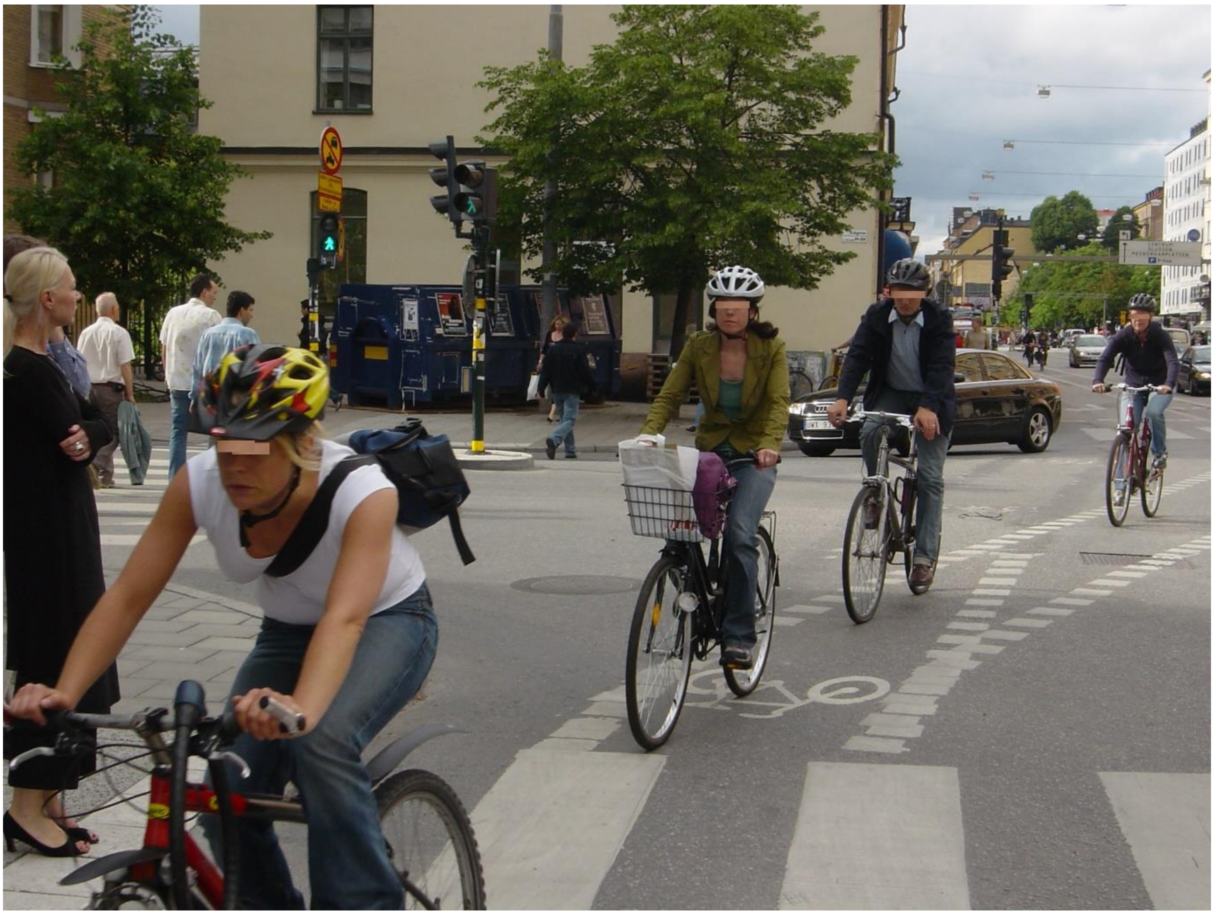

Figure 4. An inner urban setting in Stockholm with its typically about 5-6 stories high buildings in blocks and grid-like road systems. Photo: Peter Schantz.

Reference

Wahlgren, L. & Schantz, P. 2011. Bikeability and methodological issues using the active commuting route environment scale (ACRES) in a metropolitan setting. BMC Medical Research Methodology 11:6

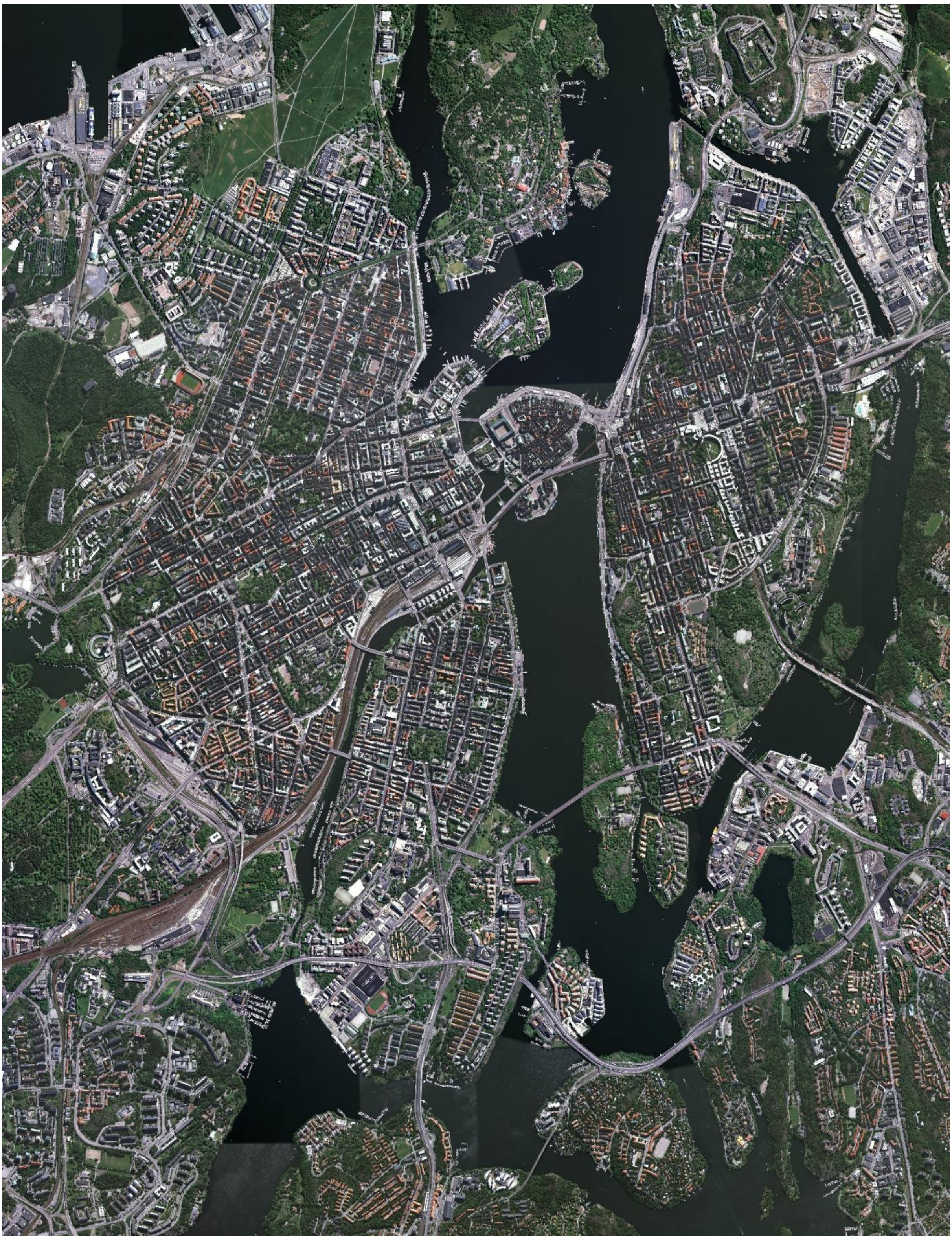

Figure 5. Aerial view over Stockholm, with the inner urban area in the middle. It is characterized by a grid-like road system, and blocks with in general 5-6 storeys high buildings. North is to the left in the image. (Copyright and permission to publish the photo is granted from The Land Survey/Lantmäteriverket, ©Lantmäteriet, Gävle, Sweden, 2011; Permission 81055230.).

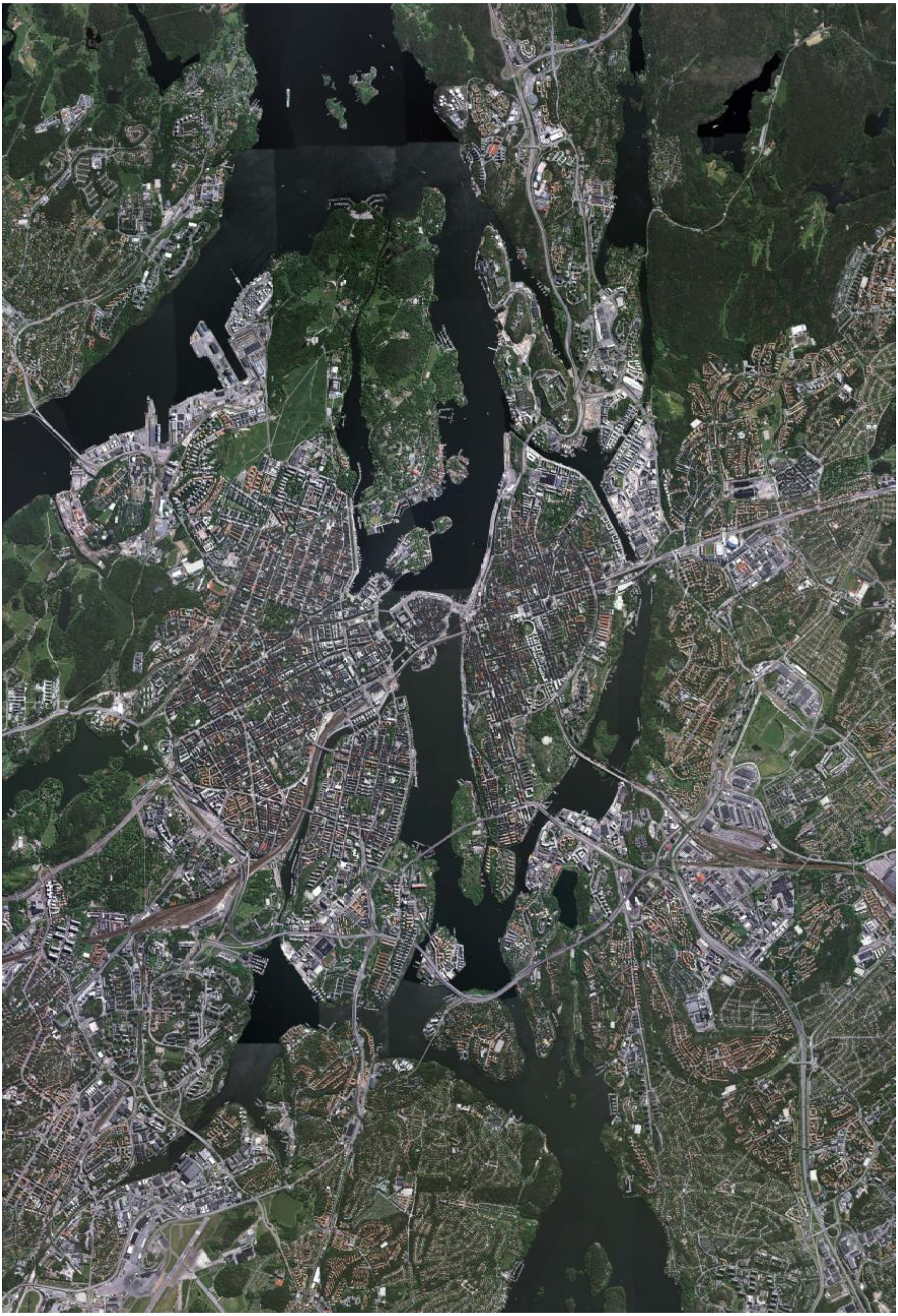

Figure 6. Aerial view over Stockholm, with the inner urban area in the middle, and various types of suburban settings around it. North is to the left in the image. (Copyright and permission to publish the photo is granted from The Land Survey/Lantmäteriverket, ©Lantmäteriet, Gävle, Sweden in 2011; Permission 81055230).
