# Supplementary material for: Perceived exertion can be lower when exercising in field versus indoors
Source: PLoS One. 2024 May 29;19(5):e0300776. doi: 10.1371/journal.pone.0300776 (PMC11135770; doi:10.1371/journal.pone.0300776)
Supplement: S4 Appendix — (PDF) [file pone.0300776.s004.pdf]

S4 Appendix. Comparisons of four studies related to physical activity, perceived exertion, and environment.

On this page a summary of higher intensity levels at different environments at equal perceived exertion (Table 1).
On the next page a detailed comparison of the studies (Table 2).

Table 1. A summary of differences in physical exercise measures in different environments at equal exertion levels. RPE = rated perceived exertion.

Study	Environment			Average relative difference in the physical exercise measures between the environments
	Indoors	Outdoors I	Outdoors II	
**Pennebaker & Lightner 1980**		Lap course	Flat cross-country trail in a wooded setting	Same levels of fatigue, higher running speeds on the cross-country trail:<br>10 %
**Ceci & Hassmén 1991**	Laboratory; treadmill, viewing a wall	Wide and curved flat path in a green and blue setting		Same RPE, higher average running speed outdoors:<br>66 %
**Mieras et al. 2014**	Laboratory; cycle trainer ergometer, viewing a wall	Paved recreation trail along a creek, with a mix of green and built-up surroundings		Same RPE, higher cycling power outdoors:<br>30 %
**Olsson et al. 2024**	Laboratory; ergometer cycle, viewing a shield	Suburban – inner urban commuting routes in a metropolitan setting		Same RPE, higher average level of %HRR and %$\dot{V}O_2$max when cycling outdoors expressed in medians:<br>23 %

Table 2. A detailed comparison of four studies on physical exercise, perceived exertion, and environment.

Study	Participant characteristics: e.g. sex, number, age	Physical activity: type and distance	Production of physical work	Type of production of physical work	Environment 1	Environment 2	Speeds, heart rates, power or exercise intensities in environment 1	Speeds, heart rates, power or exercise intensities in environment 2	Average relative difference (%) in the physical exercise measure(-s) between environment 2 and 1
Pennebaker & Lightner<br><br>1980	Male students, n = 8<br>Female students, n=5	Running<br>1800 m	Yes, in both settings	"Comfortable pace"	Outdoors:<br>lap course, 200 m x 9 laps on a flat field = 1800 m	Outdoors:<br>flat cross-country trail in a wooded setting = 1800 m	$km \cdot h^{-1}$<br>10.7	$km \cdot h^{-1}$<br>11.8	Same levels of fatigue, but higher speeds on the cross-country trail:<br>10 %
Ceci & Hassmén<br><br>1991	Males<br>n=11<br>43 yrs	Running<br>RPE 11 = 500 m<br>RPE 13 = 2000 m<br>RPE 15 = 1000 m	Yes, in both settings	RPE levels:<br>11<br>13<br>15	Indoors:<br>laboratory, treadmill, viewing a wall	Outdoors:<br>wide and curved flat path in a green and blue setting	$km \cdot h^{-1}$<br>4.3<br>6.7<br>10.4<br>$beats \cdot min^{-1}$<br>109<br>126<br>149	$km \cdot h^{-1}$<br>10.2<br>11.5<br>13.7<br>$beats \cdot min^{-1}$<br>139<br>152<br>167	Higher speeds outdoors:<br>137 %<br>72 %<br>32 %<br>Higher heart rates outdoors:<br>27 %<br>21 %<br>12 %
Mieras et al.<br>2014	Males<br>n=12<br>37 yrs	Cycling<br><br>40 km	Yes, in both settings	Instruction: "Exert as much effort as you normally would in a 40-km training ride. Try to keep your effort consistent throughout the ride. For example, don't do intervals. This effort should be perceived as the same for both your indoor and outdoor trial."	Indoors:<br>laboratory; electronically braked cycle trainer ergometer, viewing a wall	Outdoors:<br>paved recreation trail along a creek, with a mix of green and built-up surroundings	143 $beats \cdot min^{-1}$<br>24.9 $km \cdot h^{-1}$<br>163 W	152 $beats \cdot min^{-1}$<br>28.9 $km \cdot h^{-1}$<br>208 W	Same attentional focus and RPE levels (13.7) in the two environmental settings, but higher heart rates, speeds, and power outdoors:<br>6 % higher heart rates<br>16 % higher speed<br>30 % higher power
Olsson et al.<br>2024	Males<br>n=10<br>44 yrs<br><br>Females<br>n=10<br>44 yrs	Indoors:<br>ergometer cycling<br>------------<br>Outdoors:<br>commuter cycling:<br>males<br>9.6 km<br>females<br>6.5 km	Yes, in laboratory<br><br>-------------<br><br>Not outdoors when commuter cycling	3 submaximal levels on an ergometer cycle<br><br>-------------<br><br>Freely chosen commuter cycling intensities:<br><br>RPE legs / breathing:<br>males: 11.5 / 12.8<br>females: 11.5 / 12.4	Indoors:<br>laboratory; ergometer cycle, viewing a shield	Outdoors:<br>suburban – inner urban commuting routes in a metropolitan setting	Predicted field intensities based on RPE from the laboratory exercise<br><br>%HRR:<br>55.8 (breathing)/ 45.9 (legs)<br><br>$\%\dot{V}O_2max$:<br>56.4 (breathing)/ 46.9 (legs)	Measured intensities:<br><br>%HRR: 67.2<br><br>$\%\dot{V}O_2max$: 65.2	At the same RPE levels, the measured intensities in the field were higher compared to the predicted intensities. The medians of the relative differences were:<br><br>%HRR: 22.0 % (breathing)/ 30.0 % (legs)<br><br>$\%\dot{V}O_2max$: 19.1 % (breathing)/ 22.2 % (legs)
