# Supplementary material for: Perceived exertion can be lower when exercising in field versus indoors
Source: PLoS One. 2024 May 29;19(5):e0300776. doi: 10.1371/journal.pone.0300776 (PMC11135770; doi:10.1371/journal.pone.0300776)
Supplement: S5 Appendix — (PDF) [file pone.0300776.s005.pdf]

S5 Appendix. Comparisons of average exercise intensities between the entire cycle commutes versus the last 5-minute periods of the cycle commutes.

No. participants		Entire commute		Last 5-min period	
		%HRR	% $\dot{V}O_2$max	%HRR	% $\dot{V}O_2$max
**Men**	1	85.4	80.3	86.7	76.3
	2	87.3	70.5	87.4	69.1
	3	62.7	62.4	68.3	67.8
	4	76.5	74.3	75.5	68.7
	5	71.1	68.2	79.7	65.4
	6	52.4	44.3	51.5	44.0
	7	58.2	60.4	60.1	62.3
	8	53.7	59.0	58.3	60.5
	9	79.8	81.6	78.7	71.0
	10	62.6	61.2	63.2	60.9
**Women**	11	64.9	57.3	69.4	61.7
	12	66.3	51.1	70.7	55.1
	13	69.1	69.3	67.3	65.0
	14	72.1	83.4	74.5	83.2
	15	75.6	65.6	79.2	69.6
	16	69.4	60.0	77.0	71.9
	17	72.6	71.5	68.4	65.9
	18	70.0	82.8	75.2	90.0
	19	67.0	69.3	72.5	74.0
	20	61.1	64.0	65.7	65.9
	**Mean**	**68.9**	**66.8**	**71.5**	**67.4**
	**SD**	**9.3**	**10.5**	**9.1**	**9.7**
	**CI 95% low**	**64.5**	**61.9**	**67.2**	**62.9**
	**CI 95% up**	**73.2**	**71.7**	**75.7**	**72.0**
	**P-value**			**0.003**	**0.623**

Note: For this set of data, the transition periods at both the start and the end of the cycle commutes have been excluded, i.e. the transitions from resting level to exercise intensity as well as from exercise intensity back to resting level.

P-values have been calculated using the paired sample t-test.
