# Supplementary material for: Perceived exertion can be lower when exercising in field versus indoors
Source: PLoS One. 2024 May 29;19(5):e0300776. doi: 10.1371/journal.pone.0300776 (PMC11135770; doi:10.1371/journal.pone.0300776)
Supplement: Attachment — Submitted filename: Review - PONDE-D-23-12379.pdf [file pone.0300776.s007.pdf]

General commentaries

The present study aimed to investigate the relationship between indoor vs outdoor cycling RPE in habitual commuters. In order to establish this relationship, the authors collected metabolic data (HR and VO2) after a step incremental test, performed indoor, and assessed the averaged RPE response after a outdoor cycling. The results showed that the predicted RPE, based on indoor cycling, overestimates the RPE shown after an outdoor cycling bout, when normalizing by the metabolic rate (HR and VO2). Even though the topic of the present manuscript is interesting to guide public governance in terms of active commuting policies, the present methods and results have serious limitations to address the proposed goal. Please, see the commentaries below for specific issues and details.

Specific commentaries

Abstract

L.29: Since running was the example briefly discussed in the abstract, it would be better to introduce the cycling topic earlier. Some naïve reader could ask running itself was not studied, but cycling was the chosen modality.

L. 33-34: from the abstract it is not clear the reason of not using dyspnea scale for breathing. Instead the authors utilized RPE (6-20) in order to assess breathing and legs.

L. 38: from the abstract, it is not clear how exercise intensity was assessed outside of the lab.

Introduction

L. 51-56: Even though I agree about the information provided in this paragraph, it must be acknowledged that the actual methods to establish the RPE – HR relationship was the % of HR reserve. So, it would be reasonable to at least mention that %HRreserve could also be utilized.

L. 60: Note that the authors mentioned that RPE is a result of several ongoing signals, including breathing and muscle work. Thus, it would be interesting to discuss the reason for adopting RPE-breathing and RPE-leg utilized in the methods.

L. 62-66: Even though it is not the aim of the present study, someone could argue that RPE is also a result of feedforward based on expectations. For example, athletes that perform a self-paced exercise in a closed-loop task always pace themselves, partially based on the expectation to reach the finish line. It could be acknowledged here that not only afferent signals, but also some supraspinal mechanisms. Some information on the topic is given in the paragraph just below, maybe they could be merged. Please, see this meta-analysis for more details on this topic: Pharmacological Blockade of Muscle Afferents and Perception of Effort: A Systematic Review with Meta-analysis; DOI: 10.1007/s40279-022-01762-4

L. 68-73: Some studies have utilized opponents to assess the effect of external clues on self-paced exercise intensity. Please, see the following reference: Deception Improves Time Trial Performance in Well-trained Cyclists without Augmented Fatigue;
DOI: 10.1249/MSS.0000000000001483

L. 94: I assume that “three later studies” is referring to refs# 1, 7 and 9. However, only refs # 7 and 9 were explained. Would it be better to just say “given these abovementioned studies”?

L. 94-102: I see the relevance of the study, however previous data on cycling performance has already investigated the effect of external clues on self-selected exercise intensity. Please, see the following refences: Listening to Music in the First, but not the Last 1.5 km of a 5-km Running Trial Alters Pacing Strategy and Improves Performance, DOI http://dx.doi.org/10.1055/s-0032-1311581; AND Time to move beyond a brainless exercise physiology: the evidence for complex regulation of human exercise performance, doi:10.1139/H10-082

L. 100-103: Since the researchers aimed to investigate the effect of environmental clues on RPE and self-regulation of exercise intensity, I would recommend revising the references utilized in the introduction. For example, refs 11 and 10 are mostly related to surgery recovery, which is a completely difference scenario when it comes to active commuting on a bicycle.

L. 121: It is not clear the reason for utilizing % of VO2 and %HR reserve, since borg scale is set to be comparable to absolute HR responses.

L.113-126: This paragraph is methodologically right, but it does not provide the main goal and hypothesis of the current manuscript. I would strongly suggest the authors to rewrite in order to make their claim stronger and show the relevance of the work done. For example, the ACRES point was not explored in the introduction. To me, this is a key aspect of the present work. Also, have the participants rated the positive and negative appraisals for the lab environment as well? This is relevant since some people prefer cycling indoors because of better environmental control. Additionally, why only including habitual cycle commuters? This seems to be a bias in the present sample, since those participants are used to outdoor cycling but maybe less inclined for indoor cycling.

Methods

L. 143: In the introduction it was stated that the participants were “habitual cyclists”, however, the inclusion criteria was “at least once a year”. How habitual cyclists was defined for the study?

Table 1: It would be of great value for the reader to see the frequency and distance cycled throughout the participant’s commuting route. This would provide more details about the sample here utilized.

L. 167: Two or three sessions depending on which variable?

L. 171-173: The difference between RPEs here is not clear. Based on Borg’s work, RPE would be a general scale not specific to a given limb or sensation. Additionally, there are specific scales about muscle pain, discomfort and/or dyspnea that would be more useful. Has the authors asked only RPE, without specifying the sensation or limb, to the participants?

Figure 2: The figure was not displayed, so I cannot comment on the quality of the data.

Figure 3: Could the authors improve figure's 3 resolution? It is quite hard to understand the letter and values shown there.

L. 200-216: It is unclear the reason for not using the same metabolic cart for indoor and outdoor cycling. What is the bias of using those two different systems? Would that interfere in the results here presented?

L. 248-249: How steady state HR was defined here? Based on a given number over X minutes?

L. 263: It is unclear the reason for cycling at maximal power output at difference cadence.

L. 275: Where the test times similar between indoor and outdoor trials?

L. 275-277: How the authors controlled for exercise intensity in terms of power output during the outdoor cycling routes?

L. 290: This is a limitation of the study. In the indoor cycling bouts, the participants responded to the RPE at each increment on the load. However, the RPE in the outdoor bout was based on the overall session. How can the authors compare those two measurements?

L. 360: Did the authors check for data normal distribution? Additionally, even though the comparisons were made with averaged values, RPE is a ordinal variable. Thus, conventional descriptive and comparison statistics should be done in non-parametric analysis, plus presenting the results in median (range) rather than average (SD). Have the authors checked alternative approaches?

Results

Table 4: Maximal power output could be provided here.

L. 405-409: This is a central issue in the paper. It would best to comparing the overall exercise intensity of outdoor cycling with a similar metabolic strain of indoor cycling. The step increment of indoor cycling does not mimic what happened with outdoor cycling. Plus the RPE measured was related to the overall session (outdoor) rather than each exercise step (indoor).

Table 6: Did the authors controlled the outdoor cycling cadence?

Discussion

L. 459-461: Based on the current method utilized, it is not clear how the authors concluded the external clues affected the RPE responses. It would be best to state that RPE indoor vs outdoor does not correspond to the same metabolic strain. In order to test external clues, it would be best to change indoor settings while cycling rather than comparing directly to outdoor cycling, without controlling the overall route and metabolic strain as done in the indoor bouts.

L. 628: The authors could calculate metabolic thresholds in order to find the individual exercise intensity domains, since all participants performed step incremental bouts until maximal values. Additionally, the authors could calculate in which exercise intensity domain the participants cycling along their outdoor bouts. It would be very informative for matching ACSM recommendations of average physical exercise per week rather than RPE per se.
