# Supplementary material for: Perceived exertion can be lower when exercising in field versus indoors
Source: PLoS One. 2024 May 29;19(5):e0300776. doi: 10.1371/journal.pone.0300776 (PMC11135770; doi:10.1371/journal.pone.0300776)
Supplement: Attachment — Submitted filename: Reviewer.pdf [file pone.0300776.s008.pdf]

Dear authors,

Much appreciated all the effort and attention in your response. I feel that the manuscript improved, and hope the authors have the same opinion.

Nonetheless, I do have some minor commentaries (in red), which do not require changes. These are only recommendations.

Reviewer: L. 33-34: from the abstract it is not clear the reason of not using dyspnea scale for breathing. Instead, the authors utilized RPE (6-20) in order to assess breathing and legs.
Authors: *** We did not anticipate that the submaximal work rates in the laboratory and in field cycling would involve dyspnea, and therefore such a scale was not considered.
Reviewer: Ok, I guess these differences were explained in the new version, but it would be great including even more details about RPE underpinning mechanisms during exercise, with special attention to breathing and specific muscle group (legs in this case).

Reviewer: L. 60: Note that the authors mentioned that RPE is a result of several ongoing signals, including breathing and muscle work. Thus, it would be interesting to discuss the reason for adopting RPE-breathing and RPE-leg utilized in the methods.
Authors: *** Thanks for this point. We think a comment on this matter might be more suitable when we in "Methods" state our usage of this RPE division under the heading "Study design and standardization". We have now added this text, l. 170-72: The reason for this division is that RPE for a given workload is higher when it is executed by a small vs a large muscle group, whereas the central RPE, referred to as breathing, can be similar.
Reviewer: Still, some information should be added in the limitations section of the study.

Reviewer: L.113-126: This paragraph is methodologically right, but it does not provide the main goal and hypothesis of the current manuscript. I would strongly suggest the authors to rewrite in order to make their claim stronger and show the relevance of the work done.
Authors: *** Point is taken, please note the modified version of the last paragraph in Introduction. See the text on; l. 115-123.
Reviewer: Thanks for the update. As a suggestion, the figure 1 should be only mentioned once in the methods section not in the introduction.

Reviewer: Also, have the participants rated the positive and negative appraisals for the lab environment as well?
Authors: *** No. However, we secured that the laboratory environment was low on visual stimulation given that the participants looked at a uniform shield, but the perceptions evoked by it were not scored. This is now described in the Methods (l. 278-279).
Reviewer: It would be great if the participants were previously familiarized with lab environment, thus minimizing any external clue. In case you did a familiarization, this would be the place to cite it.

Reviewer: L. 171-173: The difference between RPEs here is not clear. Based on Borg's work, RPE would be a general scale not specific to a given limb or sensation. Additionally, there are specific scales about muscle pain, discomfort and/or dyspnea that would be more useful. Has the authors asked only RPE, without specifying the sensation or limb, to the participants?

Authors: *** We agree that Borg's RPE 6-20 scale was originally intended to assess the general perceived exertion. However, it is common practice at some laboratories, at least in Sweden, that the scale is used to assess the perceived exertion for breathing and musculature separately. This has long been practiced, see background in…

Reviewer: I would argue that the relevance of internal sensations induced by exercise, such as exertion, pain and dyspnea, may vary according to the intensity domain. Thus, RPE should not be used as an universal measurement of internal load during exercise.

See the manuscript below: https://doi.org/10.1152/japplphysiol.00764.2021
