# Supplementary material for: Perceived exertion can be lower when exercising in field versus indoors
Source: PLoS One. 2024 May 29;19(5):e0300776. doi: 10.1371/journal.pone.0300776 (PMC11135770; doi:10.1371/journal.pone.0300776)
Supplement: Attachment — Submitted filename: Response to reviewer no 1.docx [file pone.0300776.s009.docx]

**Authors’ response to the editor and the two reviewers in conjunction with resubmission 2**

In working with modifications given comments by reviewer no 1, matters developed.

First of all; we thought that we should add one analytical step to make our study more comparable with three other studies in the field. It now is presented in Table 9, and shows that the calculated increase in physical exercise intensity at a given RPE appears to be between 19-30% when field exercise is compared with indoor laboratory conditions.

Furthermore, we felt that now is the time to further these matters with regard to a specific weakness in previous related studies, namely the descriptions of the laboratory and external environments used.

We have therefore interviewed all corresponding authors of the previous related studies to clarify the external environmental characteristics, and to describe these issues in Supplementary Appendix files. We also create an overview of all 4 studies in an additional file.

These matters do not change any principal issues in comparison with our initial submission, but they strengthen it considerably.

All changes undertaken are shown as either “track changes”.

For specific comments to reviewer no 1´s response after resubmission 1, see below.

**Authors’ response to reviewer no 1 response after resubmission 1**

Many thanks for your further fruitful comments and recommendations that we highly appreciate.

Our comments are, given that, stated at: *******

Dear authors,

Much appreciated all the effort and attention in your response. I feel that the manuscript improved, and hope the authors have the same opinion.

Nonetheless, I do have some minor commentaries (in red), which do not require changes. These are only recommendations.

Reviewer: L. 33-34: from the abstract it is not clear the reason of not using dyspnea scale for breathing. Instead, the authors utilized RPE (6-20) in order to assess breathing and legs.

Authors: *** We did not anticipate that the submaximal work rates in the laboratory and in field cycling would involve dyspnea, and therefore such a scale was not considered.

Reviewer: Ok, I guess these differences were explained in the new version, but it would be great including even more details about RPE underpinning mechanisms during exercise, with special attention to breathing and specific muscle group (legs in this case).

*** First, just a further comment stressing why we have not made use of a dyspnea scale. Based on previous research of cycle commuting (Oja et al. 1991, se ref. in Schantz et al. 2022. [Frontiers in Public Health](https://www.researchgate.net/journal/Frontiers-in-Public-Health-2296-2565?_tp=eyJjb250ZXh0Ijp7ImZpcnN0UGFnZSI6Il9kaXJlY3QiLCJwYWdlIjoicHVibGljYXRpb24iLCJwcmV2aW91c1BhZ2UiOiJwcm9maWxlIn19) 10(911863):1-19), it could not be expected that any dyspnea would occur in our study. The results were also in line with that. The mean exercise rates in the current field cycling, undertaken of healthy participants, were about 65% of maximal oxygen uptake, and are normally not evoking any hyperventilation. The ventilatory equivalents were in line with that. Therefore, we think that introducing aspects related to the dyspnea scale would not contribute to furthering the understanding of the main aim with this study.

Regarding “but it would be great including even more details about RPE underpinning mechanisms during exercise, with special attention to breathing and specific muscle group (legs in this case).”, we cannot provide any more such information than that already stated and discussed in the ms. Indeed, given the references in this respect, mentioned in the Introduction, we noted that this field seems to clearly warrant more research in future.

Reviewer: L. 60: Note that the authors mentioned that RPE is a result of several ongoing signals, including breathing and muscle work. Thus, it would be interesting to discuss the reason for adopting RPE-breathing and RPE-leg utilized in the methods.

Authors: *** Thanks for this point. We think a comment on this matter might be more suitable when we in “Methods" state our usage of this RPE division under the heading “Study design and standardization”. We have now added this text, l. 170-72: The reason for this division is that RPE for a given workload is higher when it is executed by a small vs a large muscle group, whereas the central RPE, referred to as breathing, can be similar.

Reviewer: Still, some information should be added in the limitations section of the study.

*** Thanks. In our mind, given the comments above, we regard it to be a “strength” that we have both these kinds of RPE-measurements, and that they point in the same direction. We have added the following comment on this matter under the heading “Strengths and limitations” in the Discussion: “It is a strength that both “central” (breathing) and “local” (leg muscles) RPE were assessed. The fact that similar results were noted with these two measures strengthen the findings and point to that a general phenomenon might be underlying the differences in RPE between stationary cycling in laboratory and commuter cycling in urban and suburban commuting route environments.”

Reviewer: L.113-126: This paragraph is methodologically right, but it does not provide the main goal and hypothesis of the current manuscript. I would strongly suggest the authors to rewrite in order to make their claim stronger and show the relevance of the work done.

Authors: *** Point is taken, please note the modified version of the last paragraph in Introduction. See the text on; l. 115-123.

Reviewer: Thanks for the update. As a suggestion, the figure 1 should be only mentioned once in the methods section not in the introduction.

*** We think that this illustration is clearly important for easily understanding the analytical approach used. Therefore, it is of clear value that it is found early in the ms. Given that we think that it deserves a placing in the Introduction.

Reviewer: Also, have the participants rated the positive and negative appraisals for the lab environment as well?

Authors: *** No. However, we secured that the laboratory environment was low on visual stimulation given that the participants looked at a uniform shield, but the perceptions evoked by it were not scored. This is now described in the Methods (l. 278-279).

Reviewer: It would be great if the participants were previously familiarized with lab environment, thus minimizing any external clue. In case you did a familiarization, this would be the place to cite it.

*** Yes, they were. And we have now added that aspect after the sentence starting at l. 174: It reads: “The first test of ergometer cycle exercise in the laboratory was carried out with a purpose of familiarization”. Now it continues with: “, with regard to both all aspects of the testing, and to the laboratory environment.”

Reviewer: L. 171-173: The difference between RPEs here is not clear. Based on Borg’s work, RPE would be a general scale not specific to a given limb or sensation. Additionally, there are specific scales about muscle pain, discomfort and/or dyspnea that would be more useful. Has the authors asked only RPE,

without specifying the sensation or limb, to the participants? *** We agree that Borg's RPE 6-20 scale was originally intended to assess the general perceived exertion. However, it is common practice at some laboratories, at least in Sweden, that the scale is used to assess the perceived exertion for breathing and musculature separately. This has long been practiced, see background in…

Reviewer: I would argue that the relevance of internal sensations induced by exercise, such as exertion, pain and dyspnea, may vary according to the intensity domain. Thus, RPE should not be used as an universal measurement of internal load during exercise.

See the manuscript below: <https://doi.org/10.1152/japplphysiol.00764.2021>

*** We fully agree with you on this matter regarding a spectrum of physical activities and exercises, but given that the overall exertion in cycle commuting is rather low, we think that for our purposes RPE is a sufficient measure.
